# Utilizing AAV-mediated LEAPER 2.0 for programmable RNA editing in non-human primates and nonsense mutation correction in humanized Hurler syndrome mice

**DOI:** 10.1186/s13059-023-03086-6

**Published:** 2023-10-23

**Authors:** Zongyi Yi, Yanxia Zhao, Zexuan Yi, Yongjian Zhang, Gangbin Tang, Xiaoxue Zhang, Huixian Tang, Wei Zhang, Ying Zhao, Huayuan Xu, Yuyang Nie, Xueqing Sun, Lijun Xing, Lian Dai, Pengfei Yuan, Wensheng Wei

**Affiliations:** 1 Biomedical Pioneering Innovation Center, Peking-Tsinghua Center for Life Sciences, Peking University Genome Editing Research Center, State Key Laboratory of Protein and Plant Gene Research, School of Life Sciences, Peking University, Beijing, 100871 People’s Republic of China; 2 EdiGene Inc., Life Science Park, Changping District, Beijing, 102206 People’s Republic of China; 3 Changping Laboratory, Beijing, 102206 People’s Republic of China

## Abstract

**Background:**

The endogenous adenosine deaminases acting on RNA (ADAR) have been harnessed to facilitate precise adenosine-to-inosine editing on RNAs. However, the practicability of this approach for therapeutic purposes is still ambiguous due to the variable expression of intrinsic ADAR across various tissues and species, as well as the absence of all-encompassing confirmation for delivery methods.

**Results:**

In this study, we demonstrate that AAV-mediated delivery of circular ADAR-recruiting RNAs (arRNAs) achieves effective RNA editing in non-human primates at dosages suitable for therapy. Within a time frame of 4 to 13 weeks following infection, the editing efficiency in AAV-infected cells can reach approximately 80%, with no discernible toxicity, even at elevated dosages. In addition, when AAV-delivered circular arRNAs are systematically administered to a humanized mouse model of Hurler syndrome, it rectifies the premature stop codon precisely and restores the functionality of IDUA enzyme encoded by the Hurler causative gene in multiple organs.

**Conclusions:**

These discoveries considerably bolster the prospects of employing AAV-borne circular arRNAs for therapeutic applications and exploratory translational research.

**Supplementary Information:**

The online version contains supplementary material available at 10.1186/s13059-023-03086-6.

## Background

Adeno-associated virus-delivered cDNA therapies hold great promise, but the excessive expression of exogenous genes or high-dose AAV treatment may result in toxicity [1–3]. Although direct repair of mutations causing diseases is possible through gene-editing enzymes [4, 5], the ectopic expression of these enzymes is often associated with non-negligible problems, including extensive global off-target of genomic DNA and/or RNA transcripts [6–9], immunogenicity [10–12], oncogenicity [13, 14], and delivery hurdles due to the cargo size [15]. Therefore, utilizing intrinsic mechanisms to repair mutated genes represents a compelling alternative for gene therapy.

Programmable adenosine (A) to guanosine (G) editing could theoretically treat almost half of the genetic diseases caused by single-nucleotide polymorphisms (SNPs), including those leading to premature stop codons [16] or mutations at splicing sites [17]. As an important post-transcriptional modification, adenosine deaminases acting on RNA (ADAR) mediated RNA editing occurs widely in eukaryotic cells [18]. In this process, adenosine is deaminated to form inosine, which is subsequently interpreted as guanosine in splicing or translation mechanisms [18, 19], thereby facilitating the alteration of the genetic code. Several ADAR-dependent RNA-base editing tools have been developed [20–25]. Notably, technologies that recruit and utilize endogenous ADAR emerged as particularly promising for therapeutic applications due to the ease of delivery and safety benefits stemming from minimal off-target effects across the transcriptome [26–30].

We previously developed a programmable RNA editing technique named LEAPER (Leverage Endogenous ADAR for Programmable Editing on RNA) [27] and have recently upgraded it to LEAPER 2.0, which exhibits improved efficiency and specificity [29]. The LEAPER 2.0 technology utilizes the endogenous ADAR proteins present in cells to achieve proficient and precise RNA editing. In a mouse model, we have shown that AAV-mediated RNA editing is viable for disease treatment using LEAPER 2.0 [29]. However, the practicality of LEAPER 2.0 still needs to be evaluated in a model that closely resembles humans, such as non-human primates or human transcripts, since the activity of endogenous ADAR varies across different organs and species [31–33]. Furthermore, given that in vivo RNA editing often necessitates the use of relatively high doses of AAV [29, 30] or oligonucleotides [34], it is crucial to verify whether AAV-delivered LEAPER 2.0 can yield therapeutic benefits at doses that are clinically acceptable in non-human primates. In this study, we also endeavored to optimize the LEAPER 2.0 system further to establish robust RNA editing in non-human primates and humanized mice through AAV delivery at clinically reasonable doses.

## Results

### Circ-arRNA demonstrated effectiveness in cultured cells derived from non-human primates

**Fig. 1:**
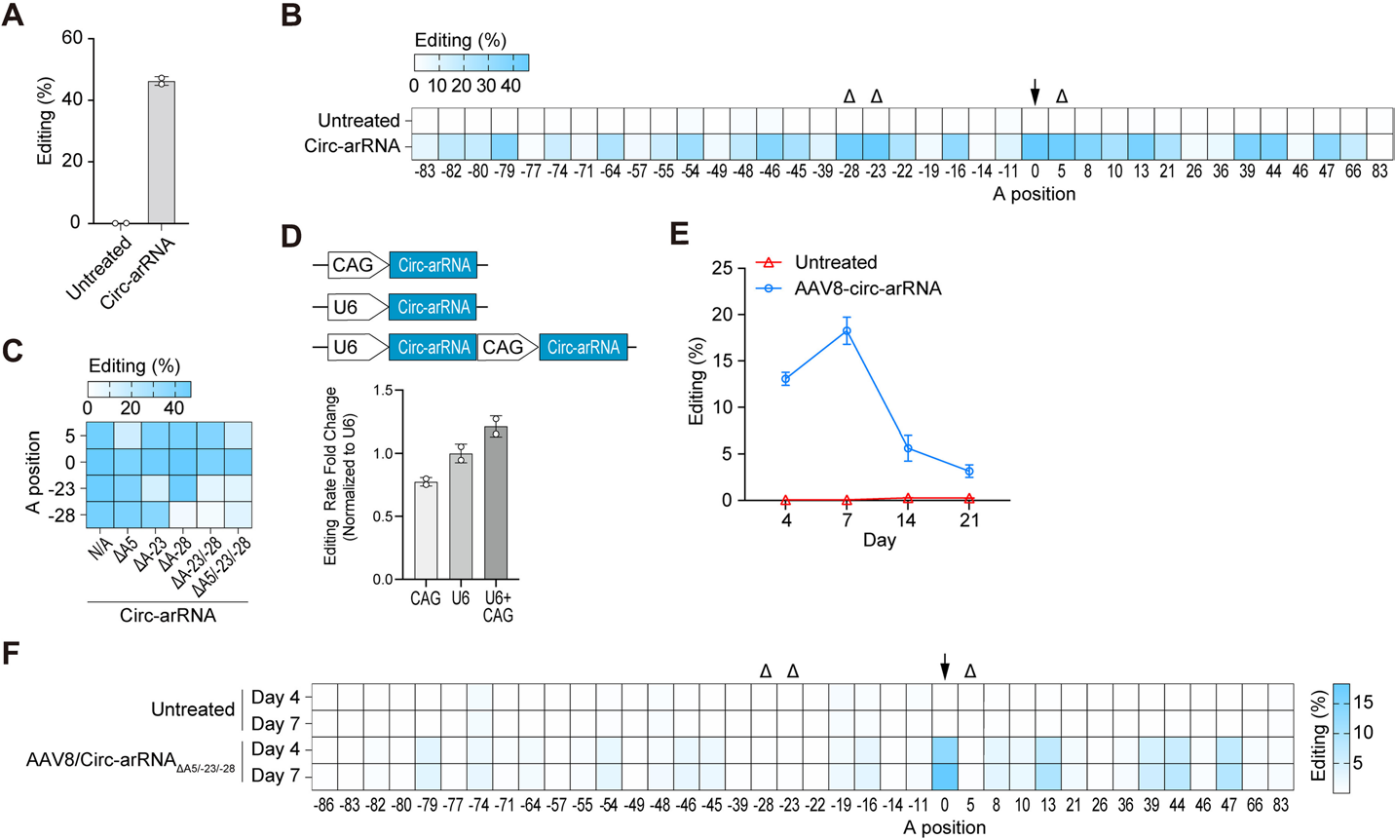
Optimization of arRNA for use in non-human primates. **A** NGS results showing the editing efficiency at target sites within *PPIA* transcripts in FRhK-4 cells derived from monkey; *n* = 2, mean ± SD. **B** NGS results showing the editing efficiency at both the target and bystander adenosine sites within *PPIA* transcripts in monkey FRhK-4 cells. The target adenosine is highlighted by a black arrow, while three severely edited bystander adenosines are denoted by black triangles; *n* = 2, mean ± SD. **C** NGS results exhibiting the editing efficiency at the target and three severely edited bystander adenosine sites of *PPIA* transcripts in monkey FRhK-4 cells. ΔA denotes circ-arRNA with corresponding uracil deletion at bystander adenosine sites;* n* = 2, mean ± SD. **D** NGS results showing the fold change in targeted editing rates when arRNA is driven by CAG (Pol II), U6 (Pol III), or a tandem combination of U6 (Pol III) and CAG (Pol II). Editing rates are normalized to the U6 promoter; *n* = 2, mean ± SD. **E** Editing efficiency of the target adenosine in primary hepatocytes from monkeys post-AAV8 infection at days 4, 7, 14, and 21 post-infection; *n* = 3, mean ± SD. **F** Editing of bystander off-target sites at days 4 and 7 post-AAV8 infection in monkey primary hepatocytes. The target adenosine is indicated by a black arrow, while three severely edited bystander adenosines are denoted by black triangles; *n* = 3, mean ± SD

To evaluate the capability of LEAPER 2.0 for precise, efficient, and long-lasting targeted RNA editing in non-human primates (NHPs), we opted to analyze *PPIA* transcripts, which were used in previous studies [29], by assessing the impact of the corresponding circ-arRNAs in monkey FRhK-4 cells through plasmid transfection. Circ-arRNAs produced approximately 50% editing of the target adenosines, along with varied editing at several adenosine sites adjacent to the target (Fig. 1A, B). As we have previously established that deletion of the nucleotide opposite the targeted adenosine can eliminate its editing, we chose three adenosine sites with substantial off-target effects and engineered the circ-arRNA to have deletions at these three respective sites. Such engineered circ-arRNA indeed significantly reduced these undesired editing while either retaining or even enhancing the on-target editing rate (Fig. 1B, C). Although the off-target editing of its bystander can be reduced by arRNA design, an excessive number of mismatches can impair the ability of arRNA to pair with the target RNA. There are currently two strategies: one is to truncate the arRNA and the other entails a more judicious design. Additionally, mutations that do not change amino acids are deemed acceptable. The current results show that the deletion design for the three sites not only reduces the off-target editing at these locations but also maintains the editing efficiency at the designated site (Fig. 1C, F).

In an effort to augment the expression of arRNA, we tested different promoters and discovered that a cassette driven by the combination of a U6 and a CAG promoter resulted in the highest editing rate (Fig. 1D). Given the small size of arRNA, it is relatively simple to combine multiple arRNAs within a single AAV vector. After delivery into primary NHP hepatocytes using AAV8, we found that the editing rate peaked up to 20% at day 7 post-AAV infection, before experiencing a sharp decline (Fig. 1E), likely due to the cell death causing the loss of AAV. The bystander off-targets were almost the same as those in FRhK-4 cells, and such off-target editing at the three critical sites was eliminated by employing a circ-arRNA specifically engineered to include the respective deletions (Fig. 1F).

### AAV-delivered circ-arRNA enables RNA editing with high efficiency in NHP

**Fig. 2:**
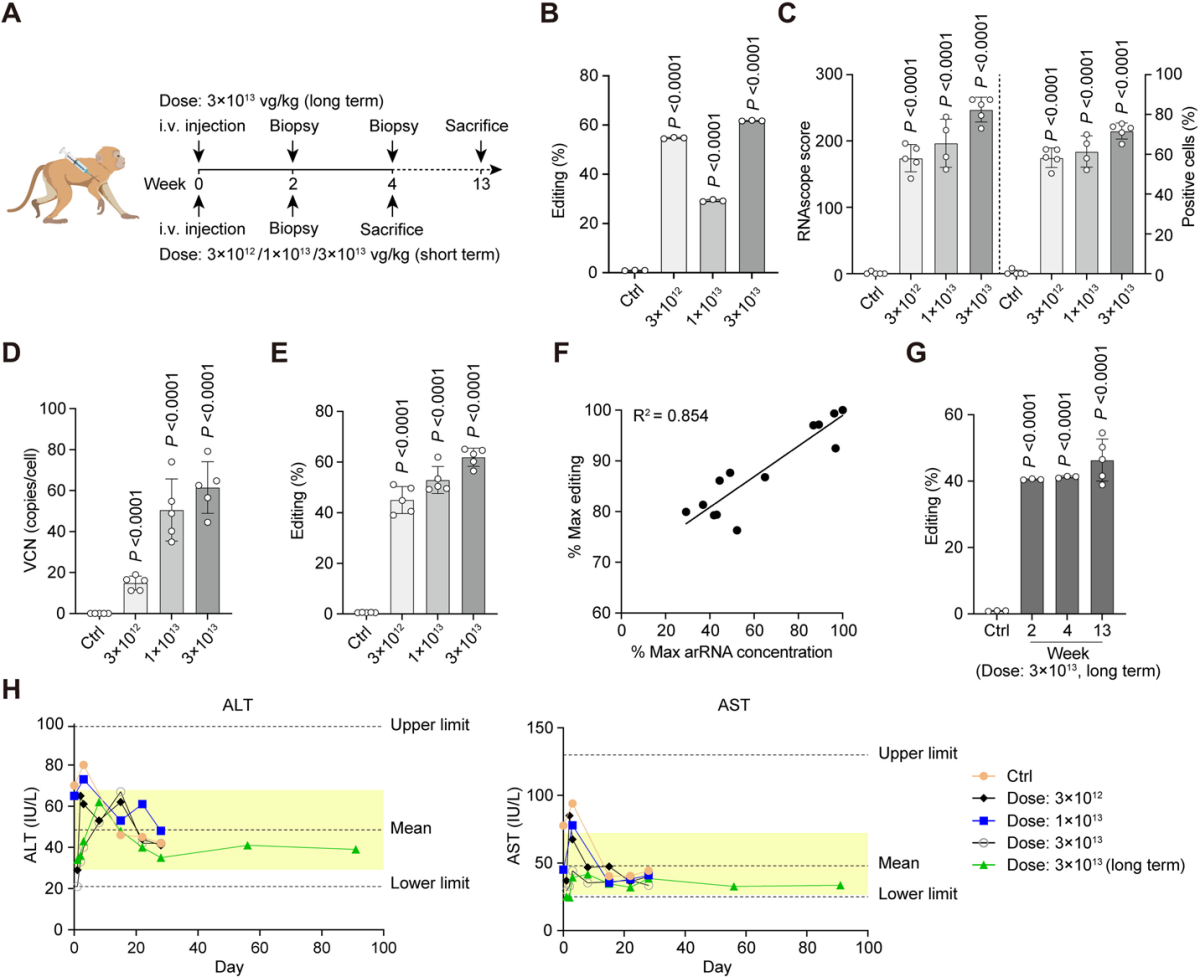
AAV-delivered circ-arRNAs facilitate highly efficient and safe RNA editing in non-human primates. **A** Diagram of the dosing schedule and sampling process. Cynomolgus monkeys were administered a one-time injection of AAV8 at three specified dosage levels. Liver biopsies were collected on the marked days, and monkeys were euthanized for comprehensive liver analysis at the specified times. **B** Editing efficiency in *PPIA* transcripts in biopsy samples at varying dosage levels at week two; *n* = 3, technical replicates, mean ± SD. **C** Editing efficiency and RNAscope scores in five distinct liver lobes were assessed by RNAscope at various dosage levels. Each dot represents data from a single liver lobe; mean ± SD. **D** Vector copy numbers in five different liver lobes measured by qPCR at different dosage levels. Each dot represents data from a single liver lobe; mean ± SD. **E** Editing efficiency of *PPIA* transcripts in five different liver lobes determined by NGS at varying dosage levels. Each dot represents data from a single lobe of the liver; mean ± SD. **F** Correlation between editing efficiency and circ-arRNA abundance across the three dosage groups. Each dot signifies data from a single liver lobe; mean ± SD. **G** Editing efficiency of *PPIA* transcripts at various time points in monkeys that received a single injection of AAV8 at 3 × 10^13^ vg/kg. Liver biopsies were collected at weeks 2 and 4, and monkeys were euthanized for comprehensive liver analysis at week 13. For biopsy samples at weeks 2 and week 4, *n* = 3, technical replicates, mean ± SD. For the week 13 sample, each dot represents data from a single liver lobe; mean ± SD. **H** ALT and AST levels post-AAV8 administration. The yellow areas indicate the typical range for healthy non-human primates. The control group consisted of cynomolgus monkey injected only with normal saline. Student’s *T* test was used for all statistical comparisons between treatment and control groups. The control group comprised two non-human primates, and each treatment group contained one non-human primate

We then examined the editing rate of circ-arRNA when delivered into NHP via AAV. Since it has been reported that AAV doses above 1 × 10^14^ vg/kg in NHP triggered strong toxicity [2], we experimented with three lower doses, namely 3 × 10^12^, 1 × 10^13^, and 3 × 10^13^ vg/kg, and chose the 3 × 10^13^ vg/kg group for a prolonged observation over 13 weeks (Fig. 2A). Biopsy samples were taken in the second week after AAV injection. We found that circ-arRNA could effectively edit the target site across all three groups, with editing rates up to 60% in the 3 × 10^12^ and 3 × 10^13^ vg/kg groups (Fig. 2B), indicating the rapid expression of circ-arRNAs. The editing efficiency failed to show a dose-dependent relationship, probably due to the variability in biopsy sampling (Fig. 2B). For the short-term groups, NHPs were euthanized 4 weeks after the injection. To achieve a comprehensive analysis of the whole organ, we divided the liver into five segments (left lateral lobe, left middle lobe, right lateral lobe, right middle lobe, and quadrate lobe) to assess editing efficiency. The efficiency of AAV transduction was evaluated with RNAscope [35] (Fig. 2C and Additional file 1: Fig. S1) and qPCR-based vector copy number (VCN) analysis [36] (Fig. 2D). Both assessments exhibited a dose-dependent trend in AAV transduction rate in the liver, with the highest rate approximating 70%. These findings suggest that AAV8 is effective for delivering circ-arRNAs to the liver.

We then examined the editing rate in the five sections of the liver. We observed that the editing rate approached ~ 50% even at the lowest dose and that the editing rate displayed a dose-dependent pattern (Fig. 2E). Considering the infection efficiency revealed by the RNAscope, we estimated that the overall editing rate for the targeted RNA was nearly 80% within the AAV-infected cells. Given that the conventional methods of quantifying RNA through reverse transcription are less accurate in evaluating circular RNAs, we devised a more specific quantitative method to measure the quantity of circ-arRNAs in the liver (refer to “Methods” section & Additional file 1: Fig. S2), and established a strong correlation between the amount of circ-arRNA and its editing rate (Fig. 2F).

To delve deeper into the sustainability and potential toxicity of editing, one non-human primate was monitored for an extended period lasting up to 13 weeks following injection. We observed that the level of targeted RNA editing remained stable over the 13-week duration without any decline (Fig. 2G).

In conclusion, the LEAPER 2.0 agents, when delivered via AAV, are capable of accomplishing effective editing not only in vitro, but also efficiently in vivo (Figs. 1 and 2), exhibiting a similar editing pattern both in vivo and in vitro. Moreover, there is a positive correlation between editing efficiency and dosage (Fig. 2F).

### Evaluation of safety concerning RNA editing through AAV-delivered circ-arRNA in NHPs

We are particularly concerned about the safety of LEAPER 2.0 for RNA editing, especially since lethal toxicity was previously observed in animals when using AAV-delivered shRNA, a different RNA modification technique harnessing an endogenous mechanism, due to the saturation of the endogenous small RNA processing pathway [37]. To evaluate safety, we diligently monitored daily clinical observations, documented body weights on a weekly basis, and collected blood samples through the femoral vein for analysis of hematology, clinical chemistry, and cytokines as per the schedule. We did not identify any noteworthy deviations between the control and experimental groups for all the parameters mentioned, even in the group under long-term observation (3 × 10^13^ vg/kg). More importantly, the levels of alanine aminotransferase (ALT) and aspartate aminotransferase (AST) activity in the experimental groups remained on par with those in the control group and were within the range indicative of a healthy NHP. Additionally, the examination of liver tissue sections revealed no evidence of liver injury throughout the experiment (Fig. 2H and Additional file 1: Fig. S3). These findings suggest that it is safe to use AAV-delivered circular arRNAs for RNA editing in NHP when administered at reasonable dosages.

**Fig. 3:**
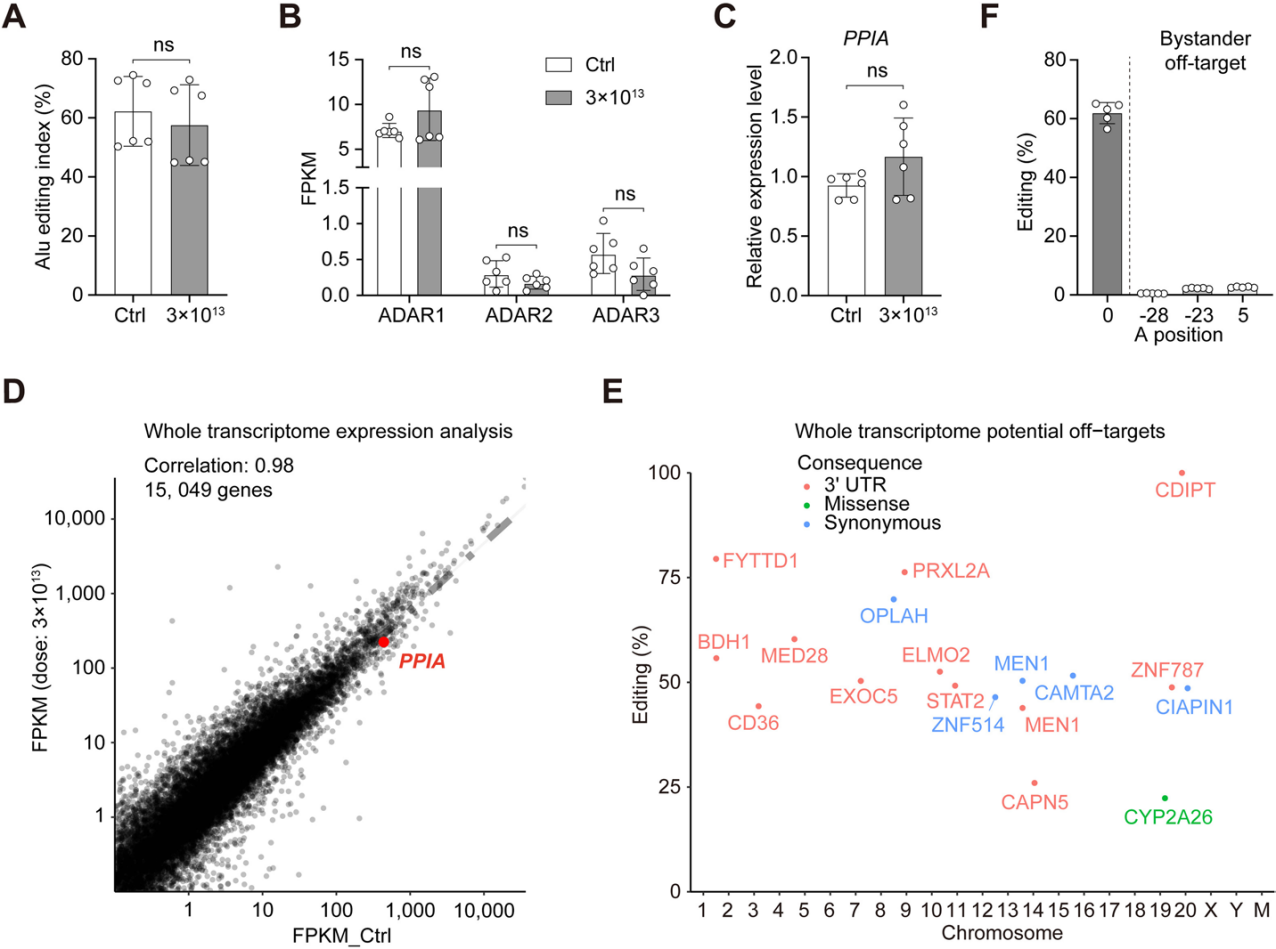
Specificity of RNA editing mediated by AAV-delivered circ-arRNA in NHPs. **A** AEI rate for the control and 3 × 10^13^ vg/kg dosage groups. Each group includes two biological replicates, and each replicate consists of 3 technical replicates; mean ± SD. **B** FPKM (Fragments Per Kilobase of exon model per Million mapped fragments) values for ADAR1, ADAR2, and ADAR3 in the Ctrl and 3 × 10^13^ vg/kg dosage groups. Each group contains two biological replicates, and each replicate includes 3 technical replicates; mean ± SD. FPKM values were calculated using the STRINGTIE tool. **C** The relative expression levels of *PPIA* transcripts measured by qPCR in the Ctrl and 3 × 10^13^ vg/kg dosage groups. **D** Differential gene expression analysis comparing the Ctrl group and 3 × 10^13^ vg/kg dosage group, based on RNA-seq data at the transcriptome level. FPKM values were calculated using the STRINGTIE tool. Pearson’s correlation coefficient analysis was used to assess the global differential gene expression. The on-targeting site (*PPIA*) is highlighted in red. **E** Transcriptome-wide analysis of potential off-target editing between the Ctrl and 3 × 10^13^ vg/kg dosage groups. The pink dots represent off-target sites located in the UTR region. The blue dots represent off-target sites causing synonymous mutations, and the green dot represents an off-target site causing missense mutation. **F** Editing rates of the target site (0) and three severe bystander off-target sites (− 28, − 23, and 5). Each point represents data from a single liver lobe; mean ± SD. Student’s *T* test was used for all statistical comparisons between the treatment and control groups

To investigate if targeted RNA editing mediated circ-arRNA has any impact on the activity of endogenous ADAR, we performed an AEI (Alu Editing Index) assay with two NHPs receiving 3 × 10^13^ vg/kg doses and a control group. The AEI reflects the averaged editing level across all expressed Alu sequences, indicating global RNA editing activity and ADAR activity [38]. The results revealed no significant difference in AEI between the experimental and control groups, indicating that the intrinsic ADAR editing activity within cells was unaffected (Fig. 3A). We also measured the expression levels of ADAR, including ADAR1, ADAR2, and ADAR3, and observed no significant changes between the experimental and control groups (Fig. 3B), consistent with AEI findings. What’s more, the RNA abundance of the targeted *PPIA* was not affected by the targeted editing (Fig. 3C, D), and the global transcriptome expression levels remained strikingly similar in the experimental and control groups (Fig. 3D). Taken together, these results indicate that circ-arRNA delivered via AAV facilitates efficient targeted RNA editing without perturbing the native ADAR activity or the global transcriptome in NHPs.

### Specificity of RNA editing through AAV delivered-circ-arRNA in NHPs

The specificity of endogenous ADAR-based RNA editing has been reported at the transcriptome-wide level in comparison with RNA editing based on overexpression of ADAR2_DD_ (ADAR2 deaminase domain) [29, 30]. We executed a similar transcriptome-wide RNA sequencing analysis in the livers of NHPs and detected only 18 potential off-target sites (Fig. 3E). Among these 18 sites in the 3 × 10^13^ vg/kg group, most were either situated in the 3′ UTR regions or resulted in synonymous mutations. Only one site, *CYP2A26*, impacted the coding sequence (Fig. 3E). The analysis of minimum free energy suggested that none of these off-target sites form a stable duplex with circ-arRNA (Additional file 1: Fig. S4). As such, it is improbable that these sites were sequence-dependent off-targets. To mitigate the bystander off-target editing, we applied a deletion strategy for three sites with the most severe bystander off-target effects and successfully eliminated all these unintended edits (Fig. 3F). Given the limited number of NHPs involved in this study, the global editing sites showed a low Pearson’s correlation coefficient (Additional file 1: Fig. S5); it would be beneficial to incorporate a larger number of NHP biological replicates in future research to further scrutinize global editing specificity.

### Optimization of circ-arRNA for treating MPS-I in a disease model harboring human *IDUA*^*W402X*^ mutation

**Fig. 4:**
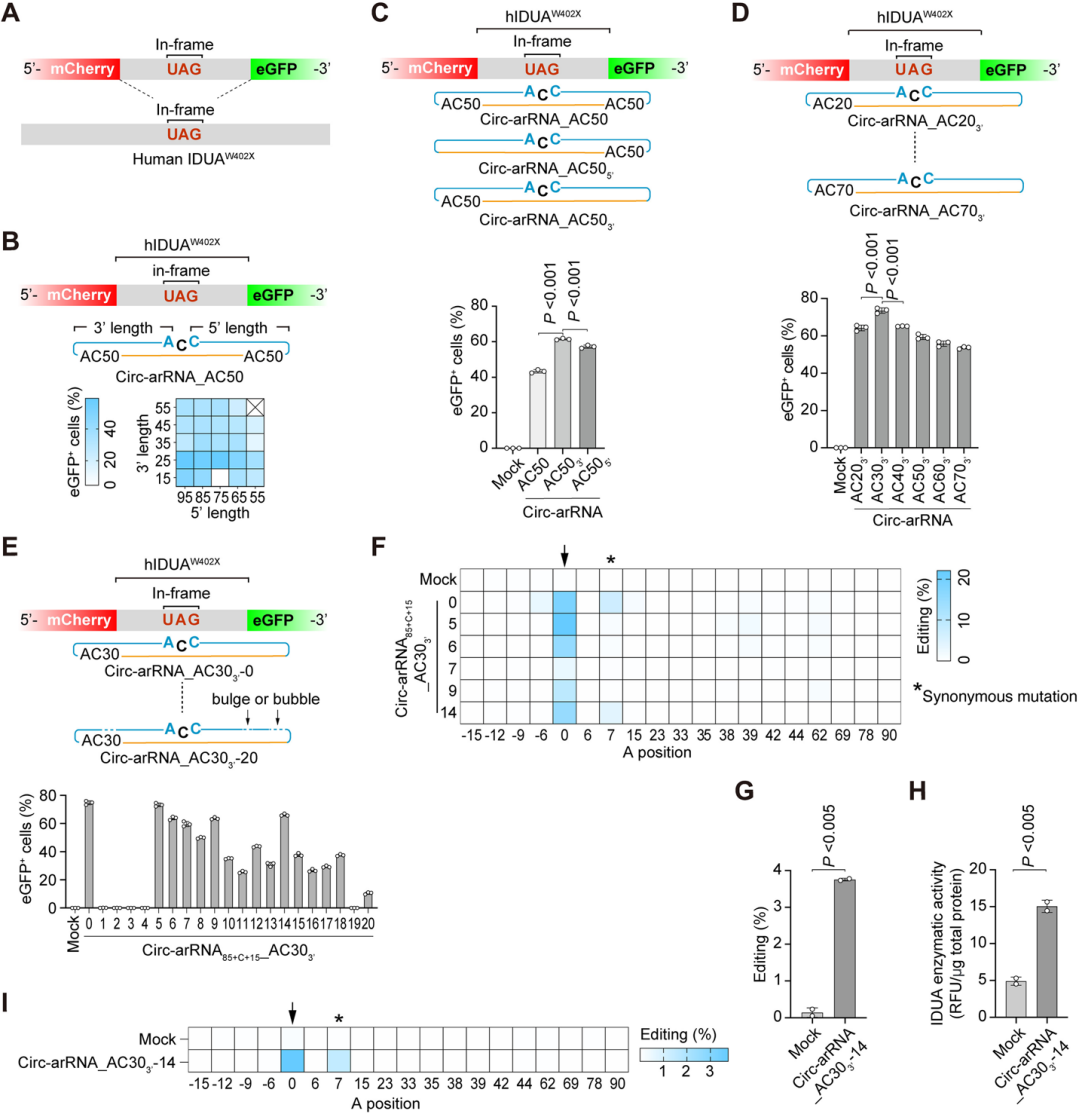
Optimization of the circ-arRNA for human IDUA^W402X^ mutation transcripts in cultured cells. **A** Diagram illustrating the human Hurler syndrome mouse model and corresponding reporter system. **B**–**D** The percentage of eGFP^+^ cells indicating the editing rate of various circ-arRNA designs targeting the reporter transcripts in HEK293T cells; *n* = 3, mean ± SD. **E** The percentage of eGFP^+^ cells and mean fluorescence intensity showing the editing rate of the reporter transcripts in HEK293T cells for 20 different versions of circ-arRNA; *n* = 3, mean ± SD. **F** NGS results showing the bystander editing for versions 5, 6, 7, 9, and 14 of circ-arRNA_85+C+15__AC30_3’_; asterisks represent synonymous mutation; *n* = 3, mean ± SD. **G** Editing rate of IDUA^W402X^ transcripts in GM06214 cells 2 weeks post-AAV-PHP.eB infection; *n* = 2, mean ± SD. **H** Measurement of IDUA protein catalytic activity using a 4-methylumbelliferyl IDUA substrate in GM06214 cells 2 weeks post-AAV-PHP.eB infection; *n* = 2, mean ± SD. **I** NGS results showing the bystander editing of *IDUA* transcripts; asterisks represent synonymous mutations; *n* = 2, mean ± SD. Student’s *T*-test was used for all statistical comparisons between the different groups

Due to the scarcity of primates’ disease models and constraints on the number of NHP that can be used, we constructed a humanized mouse model bearing the specific mutation in the human *IDUA* gene, *IDUA*^*W402X*^ (IDUA-W402X, c.1205 G > A) (Fig. 4A and Additional file 1: Fig. S6), in an effort to collect data that is more pertinent to humans. This mouse model, which harbors the human version of the coding sequence [39], mimics the human lysosomal storage disorder, Mucopolysaccharide Storage Disorder Type I (MPS-I). MPS-I is characterized by a deficiency in the enzymatic activity of α-L-idulosidase, resulting in an accumulation of glycosaminoglycan (GAG).

Given the comparable LEAPER 2.0’s editing outcomes in cultured cells and NHPs (Figs. 1C, F and 3F), we sought to optimize circ-arRNAs targeting the IDUA^W402X^ mutation using a dual-fluorescence reporter that carries the sequence encompassing the *IDUA* mutation in cultured cells. Since the bystander effect is observed within the double-stranded RNA region, it is beneficial to shorten the circ-arRNA while preserving its editing efficiency. Our prior data indicated that appending a poly(AC) linker to the arRNA could bolster the editing rate [29]. Therefore, we optimized the arRNA length and editing site employing a symmetric design with a 50-nt AC linker on both ends. The top three candidates that emerged were 85-c-25, 75-c-25, and 85-c-15 based on the eGFP reporter assay (Fig. 4B).

Considering that the W402X mutation is located in exon 9 of the *IDUA* gene and the splice acceptor site of this exon is a mere 15 bp away from this mutation, we chose 85-C-15 as a candidate arRNA for further optimization to circumvent interference with RNA splicing. We also tested whether the position of AC linker affected the effectiveness of the circ-arRNAs and found that circ-arRNAs with linkers appended only at one end (5′ or 3′) yielded superior editing efficiency compared to those with linkers on both ends (Fig. 4C). In addition, we optimized the AC linker length and observed that a 30-nt AC linker yielded the highest editing rate (Fig. 4D). Integrating these findings, we chose 85-C-15 with a 30-nt AC linker at the 3′ end, termed Circ-arRNA_AC30_3′_, for further evaluation.

To reduce the bystander off-target, we developed several strategies and engineered 20 different variants of arRNA based on Circ-arRNA_AC30_3′_ (Fig. 4E and Additional file 1: Fig. S7). Multiple bubbles or bulges exist within the designed arRNA and the target RNA sequences. According to our hypothesis, these bubbles or bulges are believed to play a pivotal role in anchoring ADAR proteins, ultimately augmenting the accuracy of the editing process (Additional file 1: Fig. S7). We observed that versions 1–4 and 19 experienced a complete loss of editing efficiency, potentially attributed to the bulges disrupting the ADAR anchoring region in these versions. In contrast, versions 5, 6, 7, 9, and 14 preserved on-target editing (Fig. 4E and Additional file 1: Fig. S7). Of these, version 14 eliminated the bystander effect, with the exception of the + 7 site (Fig. 4F). Considering that an A/G conversion at this + 7 site would not result in amino acid change, version 14 was selected for further investigation. In a parallel effort to optimize the promoter for circ-arRNA expression, we found that the 2 × U6 driver cassette was superior to the U6 plus CAG cassette for this particular sequence (Additional file 1: Fig. S8).

### Circ-arRNA delivered via AAV restored α-L-iduronidase activity in primary fibroblast cells from patients

In most MPS-I patients, the central nervous system (CNS) is severely affected, and conventional enzyme replacement therapy (ERT) could not treat the CNS because of the blood–brain barrier [40]. It has been reported that AAV-PHP.eB can penetrate the blood–brain barrier through systematic administration [41]. We then tested the potential of AAV-PHP.eB in delivering circ-arRNA to GM06214 cells derived from patient with Hurler syndrome, which contain the *IDUA*^W402X^ mutation. We observed both the correction of RNA and restoration of IDUA enzyme activity 14 days post-infection, with the editing pattern being consistent with what was seen in the reporter system (Fig. 4G–I).

### AAV-delivered circ-arRNA restored α-L-iduronidase activity in multiple organs in humanized *IDUA*^*W402X*^ mouse model

**Fig. 5:**
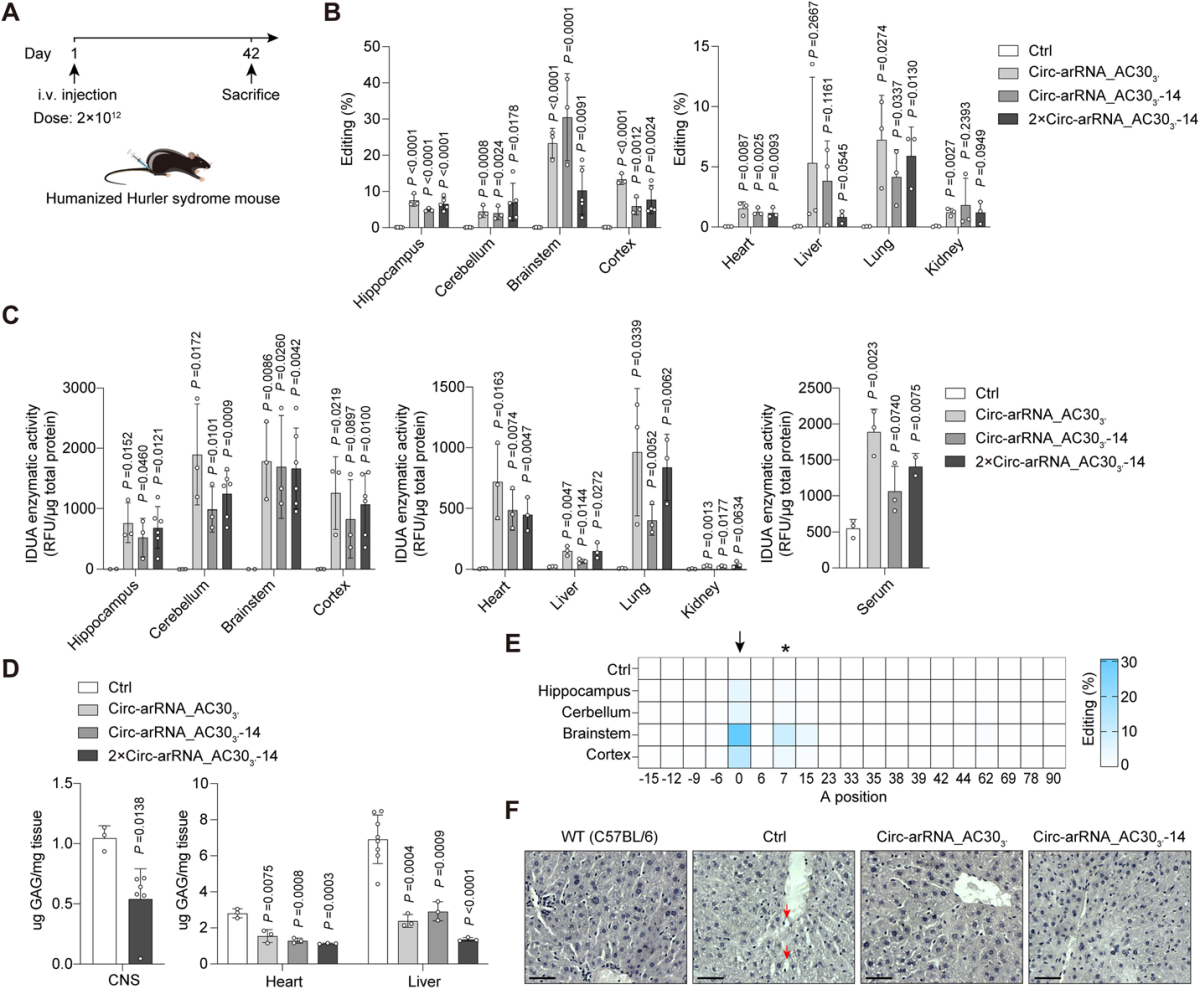
Restoration of α-L-iduronidase activity in liver and brain through AAV-delivered circ-arRNA in humanized *IDUA*^*W402X*^ mouse model. **A** Diagram showing the dosing schedule and sampling. **B** Editing rate of *IDUA*^*W402X*^ transcripts in the CNS (left) and various organs (right) in humanized Hurler syndrome mice 6 weeks after AAV-PHP.eB injection; each white dot represents an independent biological replicate, mean ± SD. **C** Measurement of IDUA protein catalytic activity using a 4-methylumbelliferyl IDUA substrate in the CNS (left), various organs (middle), and serum (right); each white dot represents an independent biological replicate, mean ± SD. **D** GAG content in tissues from the CNS (left) and various organs (right) 6 weeks post-AAV-PHP.eB infection; each white dot represents a biological replicate, mean ± SD. **E** NGS results showing bystander editing of *IDUA*^*W402X*^ transcripts in different brain regions 6 weeks after AAV-PHP.eB infection; asterisks represent synonymous mutation; *n* ≥ 2. **F** Hematoxylin–eosin staining of mice livers in different groups. Scale bar = 50 μm. Red arrows indicate foamy macrophages in the tissue due to GAG accumulation. Student’s *T*-test was used for all statistical comparisons between treatment and control groups

We then used AAV-PHP.eB for the delivery of the optimized circ-arRNA, namely U6-Circ-arRNA_AC30_3′_, U6-Circ-arRNA_AC30_3′_-14 and 2 × U6-Circ-arRNA_AC30_3′_-14, into a humanized *IDUA*^W402X^ mouse model via tail vein injection at a dosage of 2 × 10^12^ vg per mouse. Six weeks post-injection, the mice were euthanized, and tissues were collected for further analysis (Fig. 5A). The brains were sectioned into the hippocampus, cerebellum, brainstem, and cortex. We found the editing rate was detected in multiple organs, including the CNS, with rates reaching up to 30% in the brainstem (Fig. 5B). Consistent with results in NHPs, the editing rate in the CNS was correlated with the amount of circ-arRNA present (Additional file 1: Fig. S9), indicating a favorable pharmacokinetic/pharmacodynamic relationship. We then examined the IDUA catalysis activity and GAG content in mouse tissues and found that circ-arRNA significantly restored the activity of IDUA in most tissues, including the central nervous system and serum (Fig. 5C). The amount of GAG was reduced by 34% in the central nervous systems and by over 70% in the liver compared to the control group (Fig. 5D). When compared to wild-type mice (C57BL/6), the GAG levels in the liver of the treatment group were equivalent (Additional file 1: Fig. S10). The pathological examination also revealed a notable enhancement in the liver condition of the treated mice, with a reduction in the presence of foamy macrophages in tissues resulting from GAG accumulation (Fig. 5F). Similar to the reporter system, only the target site underwent editing, resulting in the conversion of a stop codon (TAG) to tryptophan (TGG), without detectable bystander off-targets affecting the coding of any other amino acids (Figs. 4F and 5E). Collectively, the delivery of optimized circ-arRNA via AAV effectively restored the lost enzymatic activity in the humanized IDUA^W402X^ mouse model. In addition, for other diseases caused by premature stop codons, which account for 11% of human inherited diseases [42], circ-arRNA holds the potential for therapeutic intervention.

## Discussion

LEAPER 2.0 [29] offers the opportunity to treat genetic diseases without altering DNA. This study establishes that delivery of circ-arRNA via AAV enables efficient RNA editing in non-human primates without severe side effects. Although Monian et al. showed that high doses of ASO effectively recruit endogenous ADAR for targeted editing in the liver of non-human primates, this is confined solely to liver tissue [34]. Naturally transcribed RNA from DNA vectors can be combined with viral vectors, particularly AAV, to enable efficient delivery to multiple organs with potential for long-term stability. However, high doses of AAV are highly associated with adverse effects, and the majority of severe side effects seen in AAV-based gene therapy in clinical trials have been reported at high doses [1–3]. Here in our study, a single dose of AAV-delivered circ-arRNA could achieve an approximate 50% editing rate in the liver of NHPs at 3 × 10^12^ vg/kg, well below the toxic dose reported (Fig. 2E). More importantly, the editing rate of AAV-delivered circ-arRNA was sustained for up to 13 weeks without any signs of decrease (Fig. 2G). In addition, our experiments conducted on humanized Hurler syndrome mice offer a demonstrative instance of the application of LEAPER 2.0. Concerning the optimization of arRNA, we initially enhanced its editing efficiency by implementing a reporting system. Subsequently, upon obtaining a highly efficient arRNA, we employed a rational design approach to introduce bubbles or bulges between the arRNA and target RNA. These structural elements are crucial for anchoring ADAR proteins, thus facilitating a more precise ADAR-mediated editing process (Fig. 4 and Additional file 1: Fig. S7). These cases serve as a significant source of inspiration for designing arRNA targeting other disease sites. Importantly, arRNA that has undergone in vitro optimization exhibits the same editing pattern in non-human primates and mice. This suggests that valuable arRNA can be readily designed in vitro, obviating the necessity for extensive animal experimentation.

## Conclusions

Taken together with our findings in NHP and humanized mice, LEAPER 2.0 offers considerable promise for clinical application in the treatment of genetic diseases, and potentially a range of other severe diseases. These discoveries considerably bolster the prospects of employing AAV-borne circular arRNAs for therapeutic applications and exploratory translational research.

## Methods

### Plasmid construction

For arRNA-expressing construct, we constructed an AAV cloning vector that included a Twister P3 U2A, 5′ ligation sequence, a 3′ ligation sequence, and Twister P1. Then, the sequences of arRNAs were synthesized and golden gate cloned into this AAV vector backbone. The arRNA sequences are listed in Additional file 2: Table S1.

For the IDUA-W402X dual fluorescence reporter, mCherry and EGFP coding sequences were synthesized with “self-cleaving” peptides P2A and T2A between them. To obtain constructs expressing IDUA gene fragments with pathogenic mutations (NM_000203.5(IDUA): c.1206G > A (p. Trp402Ter)), the G > A mutation site with 100 bp upstream and downstream sequences were synthesized (NC_000004.12 1,002647…1002,847, with pathogenic G > A mutation). This 201-bp sequence includes an 85-bp intron sequence and 116-bp exon sequence and generates a premature stop codon in the middle of it. This fragment was then inserted between P2A and T2A, and no frameshift took place. The mCherry-P2A-201nt-T2A-EGFP sequence was subsequently cloned into the pLenti-CMV-MSC-PURO backbone and packaged into Lentivirus.

HEK239T cell line which was a gift from C. Zhang’s laboratory (Peking University) was infected with the Lentivirus described above and seeded into 96-well plates with serial dilution to acquire single-cell expended colonies. The HEK239T cells have been authenticated and without mycoplasma contamination. All colonies acquired are analyzed with FACS, and the one with the highest mCherry and no EGFP intensity is selected and cultured for subsequent experiments.

### Cell culture and transfection

The HEK293T IDUA-reporter cells were cultured in DMEM/HIGH GLUCOASE Medium (Hyclone, SH302443.01) supplemented with 10% (v/v) fetal bovine serum (Vistech, SE100-11) at 37 °C with 5% CO_2_. The HEK239T IDUA-reporter cells have been authenticated and without mycoplasma contamination. FRhK-4 cells were cultured in RPMI 1640 Medium (Hyclone, SH30605.01) with 10% (v/v) fetal bovine serum at 37 °C with 5% CO_2_. Patient fibroblast (Coriell, GM06214) cells were cultured in FM (Sciencell,2301) with 15% (v/v) fetal bovine serum (Vistech, SE100-11) at 37 °C with 5% CO_2_. Rhesus Monkey hepatocytes were purchased from LONZA (LONZA, RHCP01) and cultured according to the manufacturer’s instructions. Plasmids were transfected into cells with X-tremeGENE HP DNA transfection reagent (Roche,06366546001) according to the manufacturer’s instructions. Monkey embryonic kidney cells FRhK-4 (CRL-1688, ATCC) were purchased from BeNa Culture Collection and cultured in DMEM (C11995500BT, Gibco) with 10% fetal bovine serum (SE100-011, VisTech) supplemented with 100U/mL Penicillin–Streptomycin (15,140,122, Gibco), 1 × MEM Non-Essential Amino Acids (11,140,050, Gibco), and 1 × GlutaMAX (35,050,061, Gibco) under 5% CO_2_ at 37 °C. All these cells mentioned above have been authenticated and without mycoplasma contamination.

To assess RNA editing with the IDUA-reporter cells, cells were seeded in 12-well plates (3 × 10^5^ cells/well). After 24 h, the cells were transfected with 2 μg of AAV-arRNA-BFP plasmids. Forty-eight hours after transfection, the editing efficiency was assayed by quantification of the GFP^+^ ratio. To evaluate the GFP^+^ ratio, cells were analyzed by FACS. The BFP^+^ signal served as a transfection positive cell marker, and the percentages of BFP^+^/GFP^+^ cells were calculated as the readout for editing efficiency.

To assess RNA editing efficiency in FRhK-4, 1.5 × 10^5^ cells/well were seeded in 12-well plates. After 24 h, the cells were transfected with 1 μg of AAV-arRNA plasmids. Forty-eight hours after transfection, the editing efficiency was assayed by next-generation sequencing.

AAV-PHP.eB virus (1 × 10^13^ vg/ml) was produced from PackGene Biotech. GM06214 cells were seeded in a 6-well plate (3 × 10^5^ cells/well). After 24 h, cells were infected with AAV-PHP.eB virus (1 × 10^6^ vg/cell). After 14 days, cells were assayed by the IDUA catalytic activity assay, and the editing rate was detected by next-generation sequencing.

### RNA extraction and amplicon sequencing

Total RNA was extracted with Direct-zol RNA Miniprep (R2052, Zymo Research) according to the manufacturer’s instructions. 1 μg total RNA was reverse transcribed with ProtoScript II First cDNA Synthesis (E6560L, NEB). 10 μL of ProtoScript II Reaction Mix was supplemented with 4 μL DMSO (A3672.0250, APPLICHEM), and then the template RNA and nuclease-free H_2_O were added to a total volume of 22 μl. The mixture underwent a denaturing step of 95 °C for 5 min and cooled down at 25 °C for 10 min before 2 μL of ProtoScript II Enzyme Mix was added. After this step, 24 μL of the mixture was incubated at 25 °C for 5 min and 42 °C for 1 h according to the manufacturer’s instructions. The targeted locus was PCR amplified with Q5 (M0494L, NEB) and the corresponding primers are listed in Additional file 3: Table S2. PCR products were shipped to the State Key Laboratory of Rice Biology, China National Rice Research Institute, Chinese Academy of Agricultural Sciences for next-generation sequencing.

### RNA editing analysis of targeted sites

The raw data obtained by high-throughput sequencing was subjected to quality control using fastp (v0.19.6), and the low-quality reads, the reads on adapter sequences as well as reads on sequences containing poly(G), etc., were filtered out. Subsequently, barcodes corresponding to the high-quality sequencing data obtained were split into each sample and aligned with the sequence of the amplified target region (see below for the sequence) using the BWA (v0.7.17-r1188) software, to generate a BAM file through SAMtools (v1.9) format conversion. The information obtained was statistically compared, re-ordered, and indexed. All potential RNA editing sites were detected using REDItools (v1.2.1) software, with the following parameters: with python REDItoolDenovo.py -i -f -o, after filtering out high-frequency point mutations that appeared in both control and treated samples, “(Average mutation frequency other than A > G mutation) + / − SD” was used as the threshold, and the reads of frequency value of A > G mutation at editing site above the threshold value were taken as the genuine frequency of target A to G mutation.

### NHP experiments

Rhesus monkeys were bred by the Hongfeng Experimental Animal Domestication and Breeding Center in Yulin, China. The rhesus monkeys used in this study were aged 3–5 years old and weighed 3–5 kg and were injected intravenously with different doses of AAV8, 3 × 10^12^, 1 × 10^13^, and 3 × 10^13^, respectively. As a control group, saline was injected. The physiological conditions of the animals were observed and recorded daily during the experimental period. Biological sampling was performed by liver puncture. At the end of the experiment, the animals were executed by euthanasia and the corresponding tissues were taken for further analysis. In this study, different liver lobes (containing caudate lobe, right lobe of liver, middle lobe of liver, left outer lobe of liver, and left lobe of liver) of the Rhesus monkeys were taken for the next analysis. Rhesus monkeys were fasted for at least 12 h without water prior to blood collection and testing. Blood samples (without anticoagulation) were centrifuged at 1800 × g for 10 min at 15–25 °C, and serum was separated for the aspartate aminotransferase and alanine aminotransferase assay. Aspartate aminotransferase and alanine aminotransferase activities were analyzed by IFCC method with equipment cobas 6000 c501/cobas 8000.

### Quantitative PCR for measuring the circ-arRNAs

RNA was extracted from tissues with Direct-zol RNA Miniprep (Zymo Research) according to the manufacturer’s guide. The circ-arRNAs were quantified after extraction. Briefly, the donor and the acceptor were designed as follows: the donor oligonucleotide 5′-ACAGTCCACGC-3′, the acceptor oligonucleotide 5′-AGTCGGCATGGTTCT-3′. Hybridization of oligonucleotides to the RNA was conducted in an RNase-free 0.2 ml PCR tubes on ice: 5 × Oligonucleotide annealing buffer (2 μl), 10 μM donor oligonucleotide (0.6 μl), 10 μM acceptor oligonucleotide (0.6 μl), 500 μg/ml ET SSB (2 μl), total RNA (100 ng), nuclease-free water to 10 μl. The reaction mixture was heated in a thermal cycler with heated lid (> 95 °C) to 85 °C for 2 min and then cooled to 25 °C (− 0.1 °C/s). Ligation reactions were performed as follows: 10 × Splint R Ligase Reaction buffer (2 μl); 1 μM Substrate (RNA/DNA hybrid; 2 μl); 25 U/μl SplintR® Ligase (1 μl); nuclease-free water to 20 μl. This was incubated for 30 min at 25 °C and the reaction stopped by heating at 65 °C for 20 min. We performed qPCR on the ligated product (template) using M13 universal primers (M13f: GTAAAACGACGGCCAG, M13r: CAGGAAACAGCTATGAC), according to the following reaction mix: FastStart Essential DNA Probes Master (10 μl), 250 nM of each primer, 500 nM FAM-ATGGTT + CTA + CAGTCCAC-MGB*, 2 μl template and nuclease-free water to 20 μl. Perform qPCR on a suitable real-time PCR instrument (Roche, LC96) using the following conditions: 95 °C 10 min; 40 cycles of (95 °C for 10 s, 58 °C for 10 s, 72 °C for 10 s). Samples were analyzed in triplicate for vector copy number μg DNA by the absolute quantification method using standard curves.

### RNAscope

Customized RNA probes (targeting CBH promoter) were purchased from Advanced Cell Diagnostics (ACD), and detailed information about the probes was provided in Additional file 3: Table S2. RNAscope signal was detected using RNAscope 2.5 HD Assay Red Kit per the manufacturer’s instructions available online at: https://acdbio.com/documents/product-documents. Each individual sample was analyzed with the HALOTM platform.

The RNAscope signal is binned into 5 groups (0 dots/cell, 1–3 dots/cell, 4–10 dots/cells, > 10 dots/cells, and > 15 dots/cell with > 10% of dots in clusters). Each sample is evaluated for the percentage of cells in each bin. The H-score is calculated by adding up the percentage of cells in each bin, with a weight assigned to each bin, according to the following formula: 0 × (% of cells in bin 0) + 1 × (% of cells in bin 1) + 2 × (% of cells in bin 2) + 3 × (% of cells in bin 3) + 4 × (% of cells in bin 4). H-scores will be given on a scale of 0–400. H-score = (% at 0) × 0 + (% at 1) × 1 + (% at 2) × 2 + (% at 3) × 3 + (% at 4) × 4 (H-score range is 0 to 400).

### Quantitative PCR for measuring the AAV vector copy number

Quantitative polymerase chain reaction was performed using Takara AAVpro™ Titration kit standard (cat# 6233) and the LightCycler® 96 Instrument (Roche). 50 × Primer Mix was prepared as follows: AAV Forward ITR Primer 5 μl, AAV Reverse ITR primer 5 μl, and water 15 μl. The qPCR reaction mix consisted of SYBR green Premix 12.5 μl, 50 × Primer Mix 0.5 μl, water 7 μl, and template DNA (~ 10 ng). Primers for the pAAV2 plasmid ITR were as follows: The AAV2 ITR qPCR is based on the forward primer (forward ITR primer, 5′-GGAACCCCTAGTGATGGAGTT-3′) and the reverse primer (reverse ITR primer, 5′-CGGCCTCAGTGAGCGA-3′). Thermocycler conditions were 1 cycle of 95 °C for 120 s, followed by 40 cycles of 95 °C for 5 s and 60 °C for 30 s. Samples were analyzed in triplicate for vector copy number/μg DNA by the absolute quantification method using standard curves. Preparation of the standard curve was performed following the manufacturer’s reference guide.

### NHP RNA-Seq data analysis procedures

#### Library preparation for RNA-Seq

Poly(A) + mRNA was enriched from 200 ng total RNA using a NEBNext Poly(A) mRNA Magnetic Isolation Module (New England Biolabs), and the library was prepared with the NEBNext Ultra II RNA Library Prep Kit for Illumina (New England Biolabs) following the manufacturer’s instruction. Dual indexed libraries were pooled and sequenced on an Illumina NovaSeq6000 platform with PE150 to generate 30 Gb data (~ 100 million reads) for each sample. Three replicates were included for mock and each of the treatment groups to exclude nonspecific effects.

#### Data QC

Quality control of the raw sequencing data was performed by fastp (v0.19.6) with *–detect_adapter_for_pe -g -x -q 20 -u 25 -l 50* parameters, to trim adaptor and polymer tails at the 3′ end and filter out low-quality and too short (< 50 nt) reads.

#### Mapping

The filtered reads were mapped to the rheMac10 reference genome by STAR software (v2.7.5a) in two-pass mode for subsequent variant calling, or by HISAT2 software (v2.1.0) for gene expression quantification. All alignments were sorted and indexed by samtools (v1.9) for subsequent analysis.

#### Analysis of differential gene expression

Quantification of transcriptome-wide gene expression was performed using HISAT2 (v2.1.0) and STRINGTIE (v2.1.5) software with rheMac10 reference genome and NCBI refSeq annotations from UCSC. FPKM values from STRINGTIE output were assessed for gene expression levels. For differential gene expression analysis, read counts were obtained from the HISAT2 alignment with featureCounts (v2.0.1), and the differential expression between comparison groups was further analyzed by DESeq2 (v1.36.0). Significantly differential expression genes were identified with mean FPKM ≥ 2 (for both groups) and passing the threshold of *P*_adj_ < 0.01 and |log_2_ fold change|> 2.

Global ADAR A-to-I RNA editing activity analysis with Alu Editing Index (AEI) was calculated for each sample using RNAEditingIndexer (https://github.com/a2iEditing/RNAEditingIndexer) according to Roth et al. [38] with modifications. Briefly, the qualified reads were mapped to rheMac10 reference genome with STAR (v2.7.5a) in single-pass mode and using *–sjdbOverhang 100 –outFilterMatchNminOverLread 0.95* as highlighted. The rheMac10 genome-related repeat annotation (rmsk.txt.gz), gene annotation (refFlat.txt.gz), and dbSNP were directly downloaded from the FTP site or dumped with table browser from UCSC and were further processed as instructed. For gene expression information, we prepared the expression table according to the gene expression quantification result from the STRINGTIE output in this study.

#### Transcriptome-wide editing events detection

Before variant calling, STAR aligned reads were processed following GATK best practices for calling variants in RNA-seq data (https://gatk.broadinstitute.org/hc/en-us/articles/360035531192-RNAseq-short-variant-discovery-SNPs-Indels). Briefly, the BAM alignment was fixed with AddOrReplaceReadGroups command of Picard (v2.18.23) to change group names, and PCR duplicates were marked with MarkDuplicates and further processed by SplitNCigar of GATK (v 4.1.8.0) to format RNA reads over introns, while the base recalibrating procedure was skipped.

For variant calling, GATK (v4.1.8.0) HaplotypeCaller was used to detect variants from all replicates of each comparing group in a joint calling manner. The raw variant call format files were filtered by GATK’s VariantFiltration command with the following hard filtering expression: “*QD* < *2.0 || FS* > *60.0 || MQ* < *30.0 || MQRankSum* < *-12.5 || ReadPosRankSum* < *-8.0 || DP* < *20.0 || QUAL* < *20.0*”. SNPs were selected from the filtered variants with GATK’s SelectVariants command and further filtered by bcftools (v1.9) and annotated by Ensembl Variant Effect Predictor (v102). All variants in the RefSNP database from the European Variation Archive (release 3.0) were filtered out.

For each comparison, the RNA editing level of the control group was used as a baseline, and global targets in the treatment group were ascertained by subtracting variants in the control group. This helped in identifying potential off-target sites, taking into account the consistency across all three replicates in both groups. Variants with fewer than 50 reads in total or with an editing level of < 5% in all the comparison groups were excluded. The shared global editing sites were assessed using Pearson’s correlation coefficient analysis. For the differential editing of native A-to-I editing sites, global targets with consistency across all three replicates of each group and were covered by more than 50 reads in total were selected. This was done after recognizing the reference strand and combining the data from replicates. The sites were then subjected to pairwise comparison using Fisher’s exact test, with *P* values adjusted using the Benjamini–Hochberg method. Sites with an adjusted *P* value of less than 0.01 and an absolute difference in editing level of > 10% in either direction were categorized as differentially edited sites. The novel sites that were detected only in the treatment group and were consistent across all 3 replicates in both treated animals were considered potential “off-target” sites. These were compared to those in one untreated animal (Control 1). These potential off-target sites were further analyzed through sequence homology analysis by retrieving 100 nt upstream and downstream flanking sequences from the respective overlapping transcript or genomic region. This allowed for an analysis of their similarity and duplex structure in relation to the arRNA sequence.

### Generation of humanized IDUA-W402X mice

For the mouse experiments, specific hIDUA-W402X transgene mice were generated and bred at Beijing Vitalstar Biotechnology Company Limited. Briefly, transgenic mice were generated by microinjection of the human IDUA coding cassette (CAG Promoter) carrying the W402X mutation (NM_000203.5(IDUA): c.1206G > A (p. Trp402Ter)) into the pronuclei of fertilized ova from C57BL/6 mice, which were subsequently implanted into pseudo-pregnant mice. F0 offspring were genotyped with IDUA-Tg-CF/CR, and positive ones were crossed with Idua-W392X (B6.129S-Iduatm1.1Kmke/J) female mice (Jackson Laboratory, no.017681). Genotyped positive F1 offspring were backcrossed with Idua-W392X mice again to obtain F2 Idua-W392X homozygotes (Additional file 1: Fig. S5).

### Humanized IDUA-W402X mouse AAV experiments

The experimental animals included 6- or 7-week-old humanized IDUA^W402X^ mice. Mice were housed at 18–23 °C with 40–60% humidity under a normal 12-h light–dark cycle with food and water available ad libitum under SPF (specific pathogen-free) conditions. The animal experiments were in accordance with the National Institutes of Health Guide for Care and Use of Laboratory Animals.

The titer of AAV-PHP.eB is 1 × 10^13^ vg/ml, and 200 μl of AAVs were injected into mice by tail vein per mouse. After 6 weeks, multiple mouse tissues were harvested for further assays.

### IDUA catalytic activity assay

The gathered cell pellets were resuspended and lysed with 33 μl of 0.1% Triton X-100 in 1 × PBS buffer on ice for 30 min, and the protein concentration was determined using the BCA Protein Assay Kit (Solarbio Life Sciences, PC0020). Then, 25 μl of the cell lysate was added to 25 μl of 190 μM 4-methylumbelliferyl-α-l-iduronidase substrate (Cayman, 2A-19543–500), which was dissolved in 0.4 M sodium formate buffer containing 0.2% Triton X-100 (pH 3.5) and incubated for 30 min at 37 °C in the dark. The catalytic reaction was quenched by adding 200 μl of 0.5 M NaOH/glycine buffer (pH 10.3). The fluorescence was measured at 365 nm excitation wavelength and 450 nm emission wavelength with an Infinite M200 reader (Tecan).

### Measurement of tissue GAGs

The GAG content of tissues was measured by Blyscan GAG assay kit (Blyscan, B1000). 50 mg liver or central nervous system tissue was digested with 1 ml papain extraction reagent at 65 °C for 3 h. The supernatant GAG content was assayed according to the manufacturer’s protocol.

### Pathological detection

At the end of the study, mice were euthanized, and tissues were collected and placed into 4% paraformaldehyde (PFA) for fixation and then routinely processed and embedded in paraffin blocks. The blocks were sectioned, and slides were stained with hematoxylin–eosin. Slides were evaluated by microscope.

### Statistics and reproducibility

The number of independent experiments performed in parallel is represented by *n*. Unpaired two-tailed Student’s *t*-test was implemented for group comparisons as indicated in the figure legends.

### Supplementary Information

**Additional file 1:** **Fig. S1-S10.** Supplementary figures S1-S10.

**Additional file 2:** **Table S1.** arRNA sequences used in this article.

**Additional file 3:** **Table S2.** Primer sequences used in this article.

**Additional file 4.** Review history.

## Data Availability

Raw data of off-target analysis are available as a BioProject with project identifier PRJCA019441 in the China National Center for Bioinformation-National Genomics Data Center database (GSA number: CRA012452) [43].
