# Supplementary material for: Utilizing AAV-mediated LEAPER 2.0 for programmable RNA editing in non-human primates and nonsense mutation correction in humanized Hurler syndrome mice
Source: Genome Biol. 2023 Oct 23;24:243. doi: 10.1186/s13059-023-03086-6 (PMC10591355; doi:10.1186/s13059-023-03086-6)
Supplement: Supplementary file 1 — Additional file 1: Fig. S1-S10. Supplementary figures S1-S10. [file 13059_2023_3086_MOESM1_ESM.docx]

**Additional file1**

**Utilizing AAV-mediated LEAPER 2.0 for programmable RNA editing in non-human primates and nonsense mutation correction in humanized Hurler syndrome mice**

**Zongyi Yi^1^*****, Yanxia Zhao^2^*****, Zexuan Yi^2^*****, Yongjian Zhang^2^*****, Gangbin Tang^2^, Xiaoxue Zhang^1^, Huixian Tang^1^, Wei Zhang^2^, Ying Zhao^2^, Huayuan Xu^2^, Yuyang Nie^2^, Xueqing Sun^2^, Lijun Xing^2^, Lian Dai^2^, Pengfei Yuan^2^†, Wensheng ­Wei^1,3^†**

^1^Biomedical Pioneering Innovation Center, Peking-Tsinghua Center for Life Sciences, Peking University Genome Editing Research Center, State Key Laboratory of Protein and Plant Gene Research, School of Life Sciences, Peking University, Beijing 100871, P.R. China. ^2^EdiGene Inc., Life Science Park, Changping District, Beijing 102206, P.R. China. ^3^Changping Laboratory, Beijing 102206, P.R. China.

*These authors contributed equally to this work.

†Email: pfyuan@edigene.com (P.Y.), wswei@pku.edu.cn (W.W.)

**
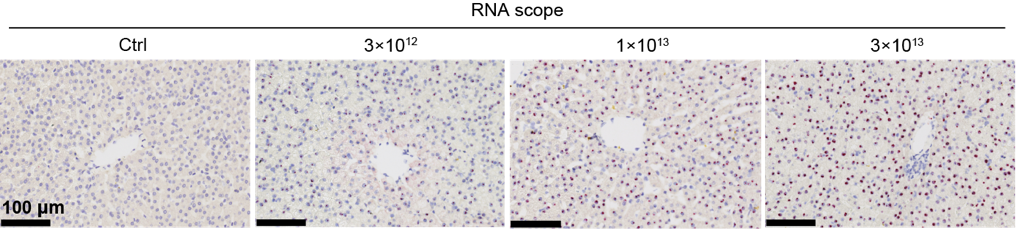
**

**Fig S1.** **The liver RNAscope images of NHPs from different dosage groups**. Scale bars, 100 μm.


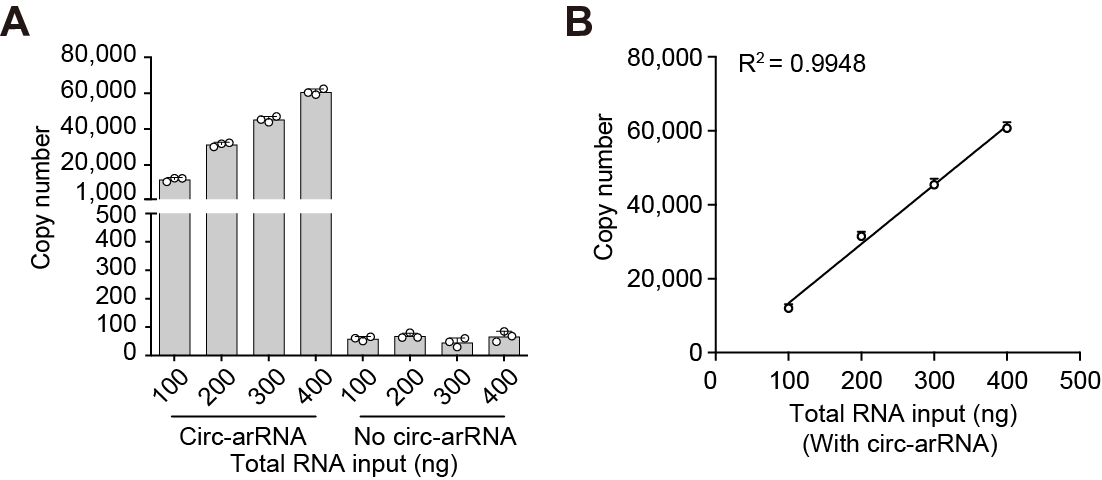


**Fig S2.** **Quantitative PCR analysis of circ-arRNA.** (A) Quantitative PCR conducted for measuring the levels of circ-arRNAs; each dot represents data from an independent biological replicate. (B) Correlation analysis between the input amount of circ-arRNA and the copy number of circ-arRNA detected. Each white dot signifies data from an independent biological sample, mean ± SD.


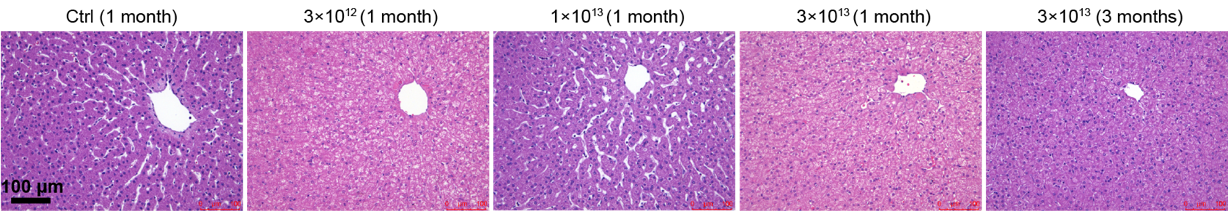


**Fig S3. Liver tissue sections from NHPs in various dosage groups.** Scale bars, 100 μm.


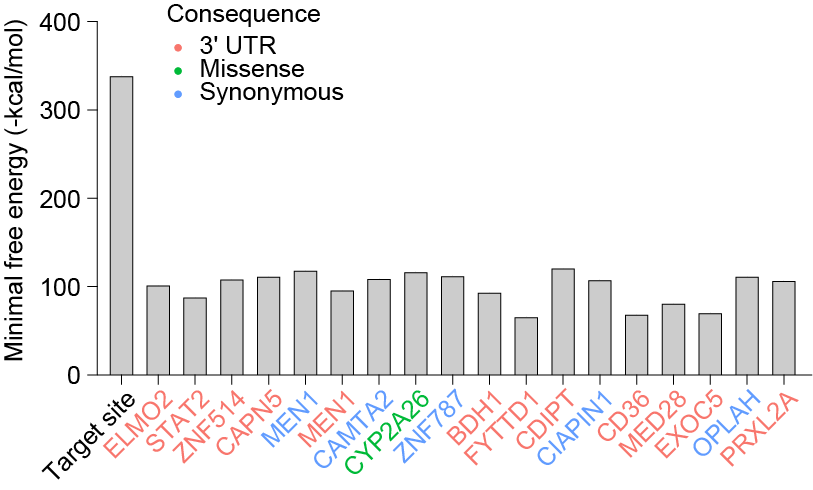


**Fig S4. Analysis of potential transcriptome-wide off-target sites.** RNAhybrid is used to perform a minimal free energy analysis of the interaction between circ-arRNA and areas of off-target sites.


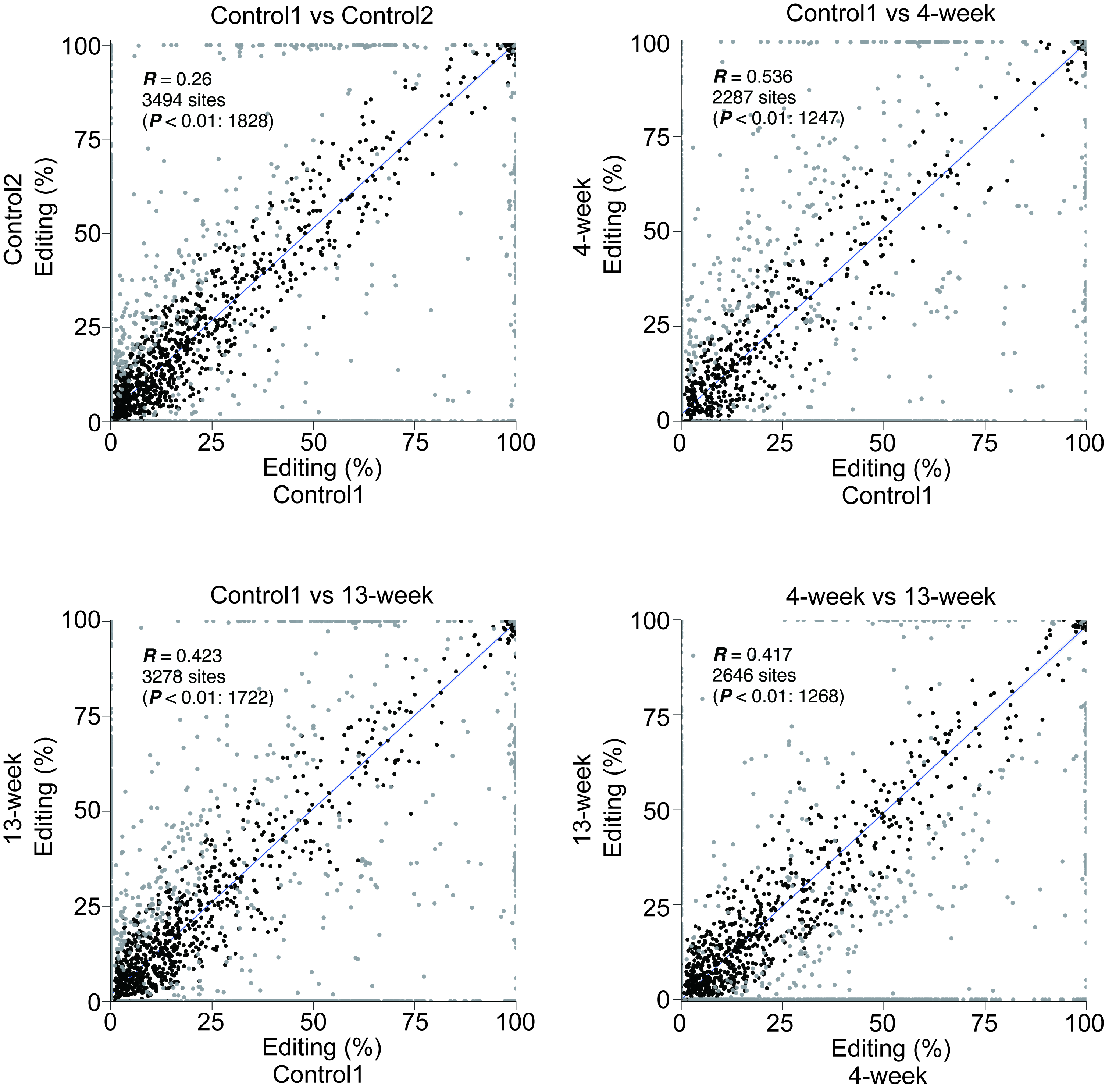


**Fig S5. Compare global editing landscape in different NHP individuals.**


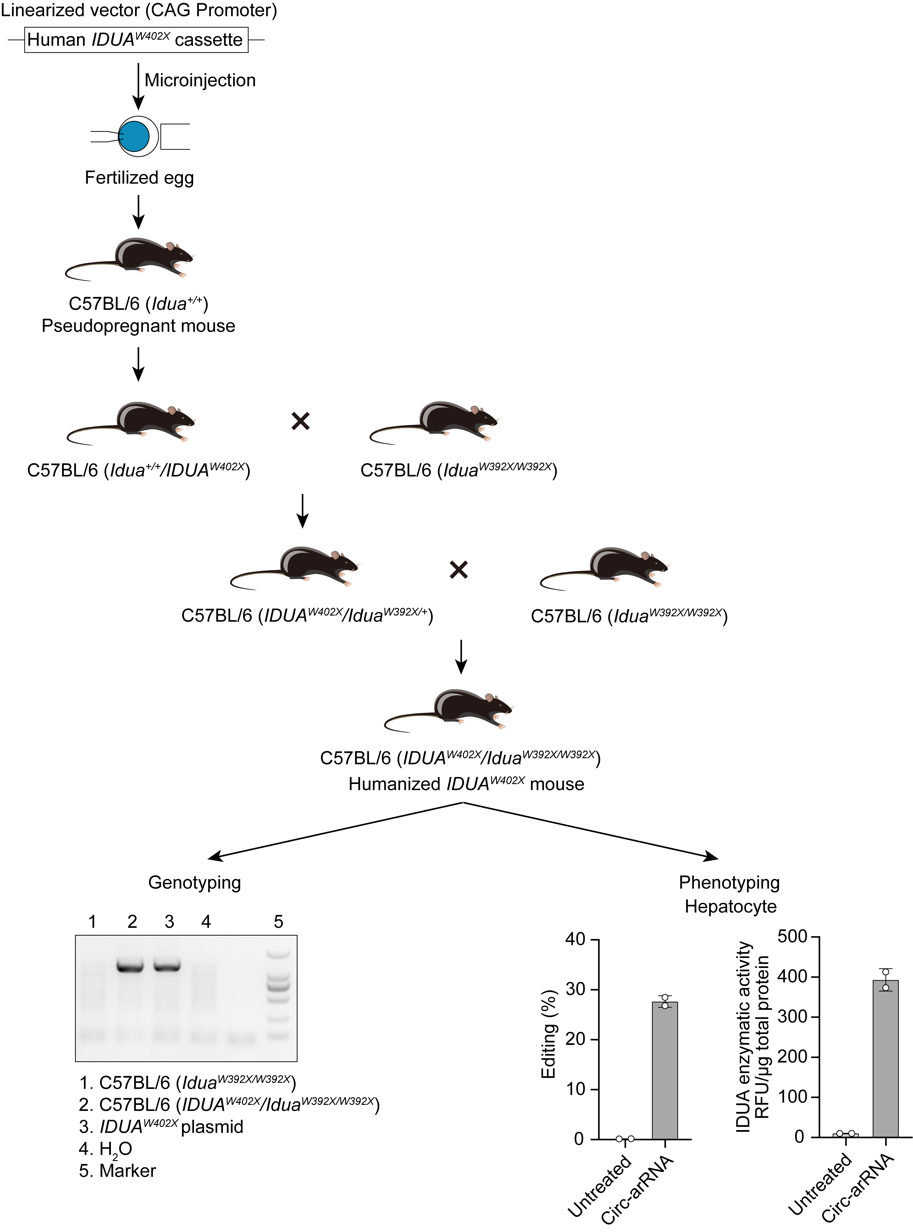


**Fig S6. Construction and genotyping of a humanized mouse model for Hurler syndrome.** Each white dot represents an independent biological replicate, mean ± SD.


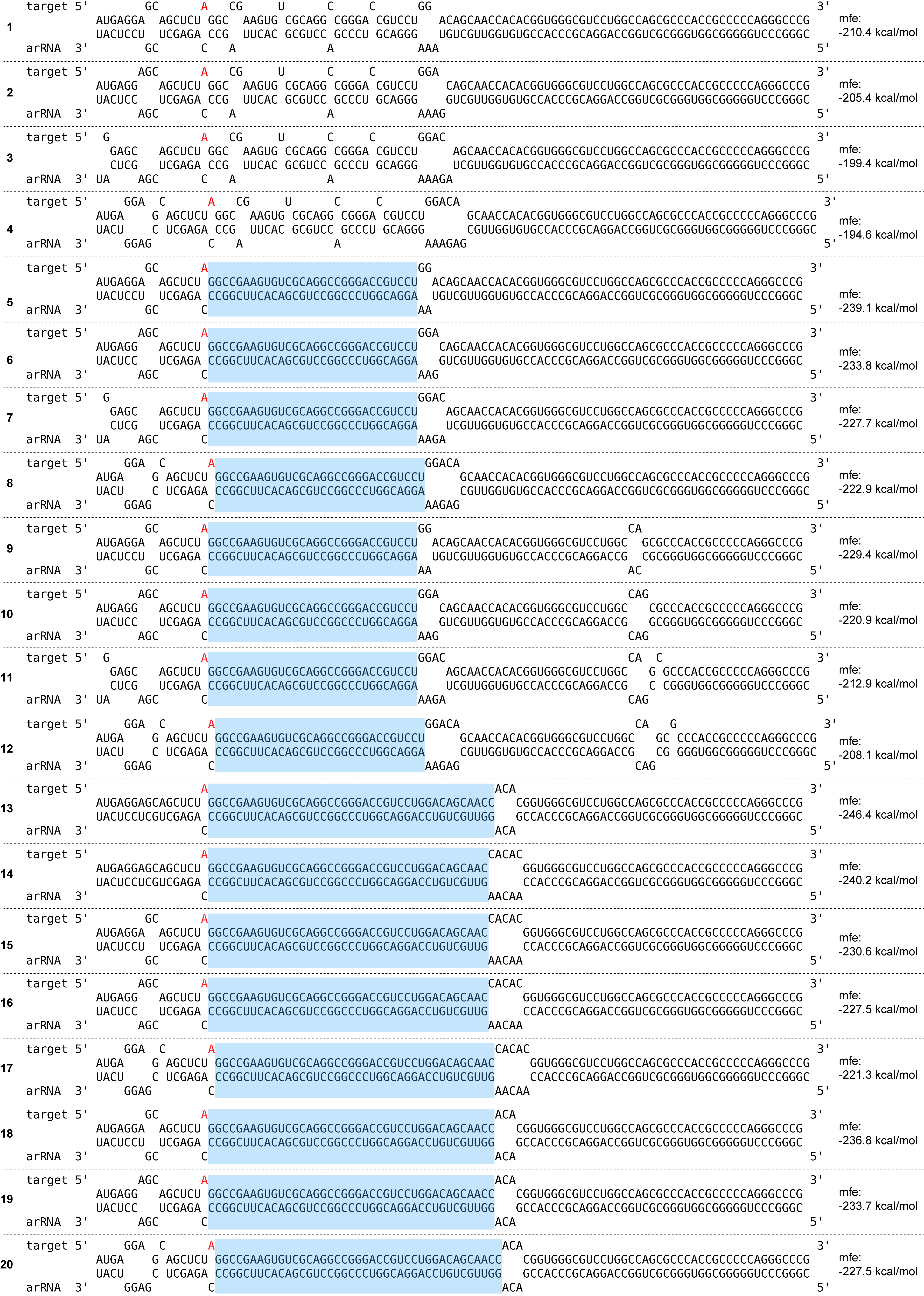


**Fig S7. RNAhybrid predicted the secondary structure of 20 different versions of circ-arRNA_AC30_3’_ in conjunction with target transcripts.** The blue background area is the potential ADAR anchoring region.


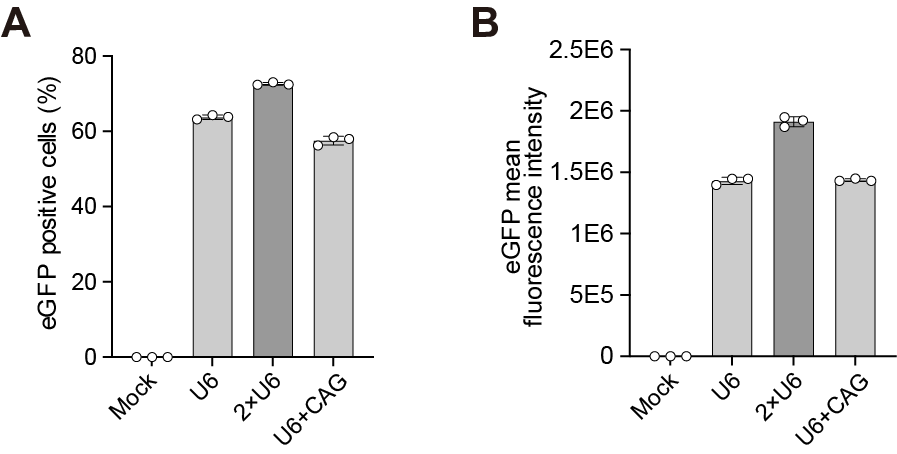


**Fig S8. Optimized circ-arRNA_AC30_3’_14_ with dual promoters in a single vector.** (A) and (B), The eGFP^+^ percentages (A) and mean fluorescence intensity (B) depict the editing rate of the reporter transcripts in HEK293T cells using different combinations of promoters. Each white dot represents an independent biological replicate, mean ± SD.


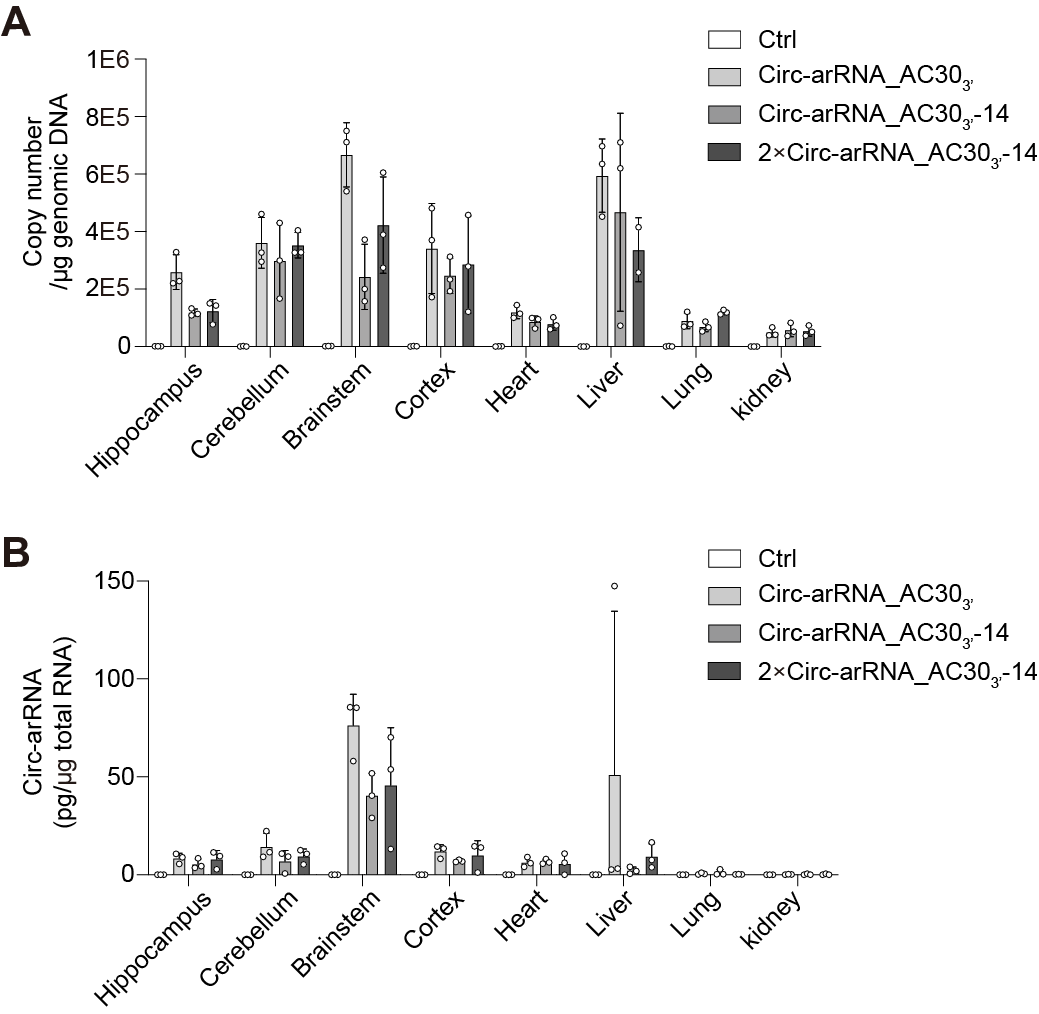


**Fig S9. Abundance of AAV-delivered circ-arRNA and AAV copy number in multiple organs of humanized Hurler syndrome mice.** Each white dot represents an independent biological replicate, mean ± SD.


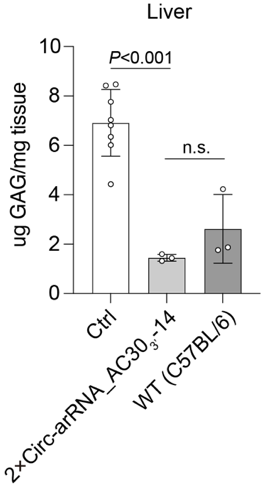


**Fig S10. GAG content in liver among different groups.** Each white dot represents an independent biological replicate, mean ± SD.
