# Supplementary material for: Utilizing AAV-mediated LEAPER 2.0 for programmable RNA editing in non-human primates and nonsense mutation correction in humanized Hurler syndrome mice
Source: Genome Biol. 2023 Oct 23;24:243. doi: 10.1186/s13059-023-03086-6 (PMC10591355; doi:10.1186/s13059-023-03086-6)
Supplement: Supplementary file 4 — Additional file 4. Review history. [file 13059_2023_3086_MOESM4_ESM.docx]

**First round of review**

**Reviewer 1**

**Were you able to assess all statistics in the manuscript, including the appropriateness of statistical tests used?**

Yes, and I have assessed the statistics in my report.

**Comments to author:**

The author's lab developed an RNA editing technique called LEAPER, and an advanced version named LEAPER 2.0, which is the endogenous mediated by adenosine deaminases acting on RNA (ADAR). This manuscript described the evaluation of optimized LEAPER 2.0 in a human-close model like non-human primates. The data highlight the potential application of the delivery of circ-arRNAs via AAV in the clinic. The claims made fit with the data provided and the manuscript is overall well-designed and well-written (Exceptions listed below).

1. Please explain the difference between Fig 1E and Fig.2B. There is very low editing (around 15%) on primary in Fig 1E, while the very high efficiency in vivo in Fig.2B.

2. Some Figs missed the statistical analysis or the methods used in Figs legend.

3. For the analysis of the off-target effect, the genomic-wide potential DNA off-targeting site is needed.

4. The definition for abbreviations (ADAR， NHP， FPKM， etc.) when they were shown for the first time.

5. The font size and weight in figs are quite small and some subsection is unreasonable (eg. Line 101-104, 148-180).

6. Language polish is required for the manuscript since many sentences are difficult to understand or not so formal.

**Reviewer 2**

**Were you able to assess all statistics in the manuscript, including the appropriateness of statistical tests used?**

Yes, and I have assessed the statistics in my report.

**Comments to author:**

In this manuscript the authors program endogenous ADAR using circular arRNAs to edit targets both in non-human primates and a humanized mouse model of disease. They provide proof-of-principle that AAV delivery of a circRNA guide can effectively edit an endogenous RNA at a tolerated dose. They further optimise their guide RNA design to limit off-target or bystander editing while enhancing on-target editing. These optimised guides are then used to edit a disease modifying allele in a humanised mouse model that results in functional changes to the targeted protein and restoration of function.

Overall, the manuscript is well written, the experimental approaches are appropriate and represent an important advance on previous work. The validation in non-human primates to show tolerance to this method of programmable editing is important for further development of this technology. Furthermore, the ability to detect editing and functional changes throughout multiple organs in the humanized mouse models, including the CNS, is commendable. Although the humanized mouse model editing was already shown somewhat in their 2022 Nature Biotech paper, there is substantially more detail here.

I have some specific concerns detailed below however I believe these can be addressed by the authors and I am overall positive about the manuscript.

Specific comments:

- In figure 1B - the authors show a great many bystander editing events and go on to prevent three of the most severe of these through deletions in the circ-arRNA but a great many remain. It is not clear how the rest of these would be prevented with the same approach without affecting the editing of the targeted adenosine as the number of mismatches increases. Is this a limitation of the design? Could the authors add some discussion around this point.

- Is the decreased editing efficiency in the NHP hepatocytes compared to the FRhK-4 cells due to decreased infection efficiency of these cells (Figure 1)?

- Please indicate in the figure legend for Figure 2 how many animals were used for this analysis.

- Is it possible that the reason for a lack of dose-dependent effect is due to saturation of endogenous ADAR? The text states that there is a dose-dependent increase in AAV transduction rate however there are no statistics on figure 2C and 2D - was this not significant between doses? With the RNA analysis shown in Figure 3 the authors should check not just for off-target effects but also for decreases in editing of endogenous sites between the control and AAV-treated animals.

- The text in lines 101-104 could be more specific - as only one NHP was kept long term it does not make sense to say "up to 3 months" the authors should be specific about the number of days. Likewise the following sentence is not very clear on why they point out no decrease for 2 biopsies at weeks 2 and 4 but not 13 weeks?

- For figure 3D, it would be more standard to represent this data as a Volcano plot to see how many statistically significant gene changes there are between control and treated animals. The correlation plot does not allow assessment of how significant the effects on gene expression globally are. It would also be interesting to see whether interferon-stimulated genes are activated due to reprogramming of ADAR and loss of editing at endogenous immunogenic sites (or whether ADAR expression is changed).

- For the optimization of the 5' and 3' lengths in the circ-arRNA, could the authors comment on why these lengths were chosen and why the 5' length needs to be longer than the 3' length.

- Since the Humanized Hurler syndrome mouse is not used in Figure 4 it would be clearer to move the illustration in part A to Figure 5.

- The editing percentage in the GM06214 cells is very low compared to other experiments. Could the authors comment on this? Is this due to the cell type or the AAV vector used? For the IDUA enzymatic activity shown it would be helpful to have a WT control to be able to compare how significant this improvement in enzyme function is.

- Statistical analysis on figures 4 and 5 is completely absent which drastically limits the interpretation of the findings and how meaningful the observed changes are particularly in the Hurler-syndrome mouse model. This should be added.

- No WT (or heterozygous) control is included in the analysis of Humanized Hurler syndrome mouse model which would be very helpful in the interpretation of how significant the improvement in IDUA gene function is relative to WT. While the lack of this does not negate the findings it does limit interpretation. The authors could compare to other data using these assays with WT mice and comment on how substantial the changes are. Furthermore, there is no description of the phenotype of this mutation in mouse - are they sick, is survival affected? While enzymatic activity is improved, does this improve the overall health of the animals? How does this model compare to phenotypes in humans with this mutation?

- Is the 2x Circ-arRNA used in Figure 5 using the 2x U6 promoters shown in Fig S7? This should be explicitly stated. There is no interpretation of the comparison of the different circ-arRNAs used in this figure in the text. This should be added to the discussion of this figure.

Minor comments:

1. On the first use of an acronym they should be defined - including arRNA, LEAPER and NHP

2. The introduction would benefit from a brief introduction to the LEAPER method.

3. The manuscript should be checked for grammar. Some specific minor corrections noted here:

a. line 54 - remove "a" before the word robust

b. line 71 - change delivered to delivery

c. line 107 - change "to LEAPER" to "of LEAPER"

d. line 172 - "test" to "testing"

e. line 178 - typing error in parallel

f. line 187 - I believe the authors mean systemic not systematic?

g. Line 806 - breaded to bred.

4. What does CRO stand for in the Figure 2 figure legend?

**Reviewer 3**

**Were you able to assess all statistics in the manuscript, including the appropriateness of statistical tests used?**

Yes, and I have assessed the statistics in my report.

**Comments to author:**

RNA editing holds promise as a therapeutic strategy that enables temporary and non-heritable edits. The utilization of systems that deliver a single gRNA to recruit endogenous ADARs has great therapeutical potential. In this study, the authors demonstrate the effectiveness of AAV-delivered circular arRNAs for achieving RNA editing in non-human primates at therapeutic doses. Furthermore, AAV-delivered circular arRNAs in a humanized Hurler syndrome mouse model rescued IDUA enzymatic activity in multiple organs. While this work provides preliminary evidence supporting the feasibility of AAV-based gRNA delivery in therapy and translational research, a number of concerns need to be addressed to substantiate their claims.

1. The NHP experiments have a key flaw as the authors only tested a single site with a TAG motif, which is known to be the easiest to edit, to showcase the potential of cir-arRNA for RNA editing in the NHP liver. To establish the efficacy of AAV-mediated LEAPER 2.0 for editing in NHP liver, it is crucial for the authors to test additional sites, especially those with non-TAN motifs, to demonstrate that efficient editing can be achieved beyond the TAG motif.

2. From Fig. 1F, it appears that the arRNA shows considerable off-target editing at positions 13, 39, 44, and 47. It would be important to investigate the editing statuses of these sites in the NHP liver. If these sites are also edited in NHP liver, the authors should use a deletion strategy to remove these unwanted edits in vivo. Since deletions generally decrease the dsRNA structure between arRNA and its target, it is crucial to demonstrate that this approach maintains on-target editing while removing off-target edits in this case.

3. Figure 3 requires a more detailed analysis of global off-targets.

a) Editing sites with >5% or 10% editing levels may have phenotypic effects. Thus, the authors need to accurately describe the landscape of off-targets using lower editing level cutoffs.

b) The method section for off-target analysis is not clear. The authors need to define how a site is considered an off-target. Additionally, although the method section describes the analysis of differentially edited sites, there are no related results in the results section.

c) A comparison of editing levels of individual sites between control and edited NHP samples is necessary to estimate the impact of AAV-delivered arRNA on the global editing landscape.

d) Further analyses are needed for the off-target sequences to determine possible reasons. For example, investigating the sequence similarity among the off-target regions and examining if short duplex formation occurs between arRNA and off-target sequences.

4. Fig. 4A: There is no description of the transgenic mice used. The authors should provide information on the promoter used and the expression profile of the hIDUAw402x gene, and whether it is similar to the original gene.

5. Fig. 5. The authors should measure the IDUA enzymatic activity and GAG content of wild-type mice as controls. This will provide a baseline for evaluating the extent to which RNA base editing rescues these molecular markers.

6. It is crucial for the authors to show that arRNA-based RNA editing rescues the phenotype of the disease model. Since IDUA mutant mice are known to exhibit bone abnormalities, craniofacial abnormalities, and reduced lifespan, the authors should provide evidence that RNA editing leads to the rescue of these phenotypes. Additionally, histopathological evidence of reduced lysosomal storage in various tissues in the edited samples should be presented.

Minor comments

1. Figure 3D. Please label the differentially expressed genes and analyze the functions of the upregulated and downregulated genes, respectively.

2. RNA-seq data generated in this study should be deposited and released in a publicly accessible repository for transparency and reproducibility.

**Authors Response**

**Point-by-point responses to the reviewers’ comments:**

Reviewer #1: The author's lab developed an RNA editing technique called LEAPER, and an advanced version named LEAPER 2.0, which is the endogenous mediated by adenosine deaminases acting on RNA (ADAR). This manuscript described the evaluation of optimized LEAPER 2.0 in a human-close model like non-human primates. The data highlight the potential application of the delivery of circ-arRNAs via AAV in the clinic. The claims made fit with the data provided and the manuscript is overall well-designed and well-written (Exceptions listed below).

*Response: Thank you for your encouraging comments.*

1. Please explain the difference between Fig.1E and Fig.2B. There is very low editing (around 15%) on primary in Fig 1E, while the very high efficiency in vivo in Fig.2B.

*Response: Thank you for posing your question. In Fig. 1E, we conducted an in vitro infection of monkey liver cells using AAV8, while in Fig. 2B, we performed an in vivo infection of monkey liver through AAV8. Although the same AAV serotype was used, we attribute the higher efficiency in vivo to several factors. First, the condition of hepatocytes cultured in vitro might not be as optimal as in the natural environment of the body, possibly resulting in a lower infection efficiency. Second, hepatocytes cannot undergo cell division in vitro, and as they deteriorate, the editing efficiency correspondingly diminishes. Lastly, the quantity of AAV applied differed in the two experiments. We believe that these factors contribute to the disparity in editing efficiency observed.*

2. Some Figs missed the statistical analysis or the methods used in Figs legend.

*Response: Thank you for your suggestion. We have added the statistical analysis in Figs and the corresponding methods in Figs’ legends.*

3. For the analysis of the off-target effect, the genomic-wide potential DNA off-targeting site is needed.

*Response: Based on current knowledge, it is understood that ADAR lacks the ability to edit DNA. Consequently, in principle, when arRNA is expressed, there should not be any off-target DNA editing due to ADAR's inability to act on DNA.*

*Nevertheless, we performed whole genome sequencing on brain and liver tissue samples (the liver typically has high AAV8 distribution, whereas the brain has minimal distribution due to the blood-brain barrier, making it suitable non-target control within the same individual). We extracted genomic DNA from ~25 mg of frozen tissue of two monkeys that had been injected with AAV, using the DNeasy Blood & Tissue Kit (QIAGEN) as per the manufacturer’s instructions. We then constructed whole genome libraries using the MGIEasy PCR-Free DNA Library Prep Kit (MGI) according to the DNBSEQ library preparation protocol.*

*Reviewer Figure 1. Relative contribution of each of the 96 trinucleotide changes between the somatic mutation profile of the two AAV-treated animals (upper and middle panels), and the difference between these profiles (lower panel) for the two animals. The residual sum of squares (RSS) and the cosine similarity between the two mutational profiles are indicated.*

*In summary, brain and liver samples were sequenced with an effective coverage ranging from 34.6× to 42.6× after quality filtering and removal of duplicates from the final alignment. In* *total, 320,652 and 291,933 somatic mutations were detected in the samples treated for 4 weeks and 13 weeks respectively, while only 40 somatic mutations were common to both samples (Reviewer Figure 1). Analysis of the base substitution mutation spectrum showed that two samples had highly similar mutation patterns, suggesting that these somatic mutations were normal background mutation events. A BLAST search conducted with the 40 common sites and all somatic sites from the two samples failed to yield any significant hits, and analysis of the flanking sequences showed that there was no homology between any of any of the 40 common mutation sites and the arRNA sequence. Collectively, these results indicate that there were no off-target events at the DNA level in monkeys injected with a high dose of AAV.*

4. The definition for abbreviations (ADAR， NHP， FPKM， etc.) when they were shown for the first time.

*Response: Thank you for your suggestion. We have added full name when they were shown for the first time.*

5. The font size and weight in figs are quite small and some subsection is unreasonable (eg. Line 101-104, 148-180).

*Response: Thank you for your suggestion. We have changed the font accordingly.*

6. Language polish is required for the manuscript since many sentences are difficult to understand or not so formal.

*Response: Thank you for your suggestion. We have carefully polished our manuscript.*

Reviewer #2: In this manuscript the authors program endogenous ADAR using circular arRNAs to edit targets both in non-human primates and a humanized mouse model of disease. They provide proof-of-principle that AAV delivery of a circRNA guide can effectively edit an endogenous RNA at a tolerated dose. They further optimise their guide RNA design to limit off-target or bystander editing while enhancing on-target editing. These optimised guides are then used to edit a disease modifying allele in a humanised mouse model that results in functional changes to the targeted protein and restoration of function.

Overall, the manuscript is well written, the experimental approaches are appropriate and represent an important advance on previous work. The validation in non-human primates to show tolerance to this method of programmable editing is important for further development of this technology. Furthermore, the ability to detect editing and functional changes throughout multiple organs in the humanized mouse models, including the CNS, is commendable. Although the humanized mouse model editing was already shown somewhat in their 2022 Nature Biotech paper, there is substantially more detail here.

I have some specific concerns detailed below however I believe these can be addressed by the authors and I am overall positive about the manuscript.

*Response: Thank you for your encouraging comments.*

Specific comments:

-	In figure 1B - the authors show a great many bystander editing events and go on to prevent three of the most severe of these through deletions in the circ-arRNA but a great many remain. It is not clear how the rest of these would be prevented with the same approach without affecting the editing of the targeted adenosine as the number of mismatches increases. Is this a limitation of the design? Could the authors add some discussion around this point.

*Response: Thank you for your input. Indeed, Although reducing off-target editing of bystander regions through arRNA design is essential, but as you rightly pointed out, too many mismatches can hinder arRNA’s ability to hybridize with the target RNA. The two strategies you mentioned, namely shortening the length of arRNA and employing more sophisticated design considerations, are promising. Furthermore, tolerating mutations that do not change the amino acid sequence may be an acceptable trade-off in certain cases. The current results, which demonstrate that deletion designs at the three sites not only reduce off-target editing at these locations but also maintain the editing efficiency of the target site, are encouraging. The pursuit* *of more refined designs to reduce bystander off-target editing continues to be a worthwhile endeavor.*

-	Is the decreased editing efficiency in the NHP hepatocytes compared to the FRhK-4 cells due to decreased infection efficiency of these cells (Figure 1)?

*Response: Thank you for raising this question. You're correct in pointing out that the two different methods employed for introducing arRNA into NHP hepatocytes and FRhK-4 cells can lead to variations in editing efficiency. In the case of NHP hepatocytes, we utilized AAV8 infection, whereas for FRhK-4 cells, we employed plasmid transfection. These distinct methods can result in different levels of arRNA expression in the cells, which is likely the underlying reason for the observed differences in editing efficiency between these two cell types.*

-	Please indicate in the figure legend for Figure 2 how many animals were used for this analysis.

*Response: Thank you for your suggestion. We have added the number in legend. Please refer to the table below for the corresponding numbers (Reviewer Table 1).*

Reviewer Table 1. Number of animals used between groups.

| Duration of Injection | Dose | Number of animals |
| --- | --- | --- |
| / | PBS | 2 |
| 4-week | 3×10^12^ vg/kg AAV8-arRNA | 1 |
| 4-week | 1×10^13^ vg/kg AAV8-arRNA | 1 |
| 4-week | 3×10^13^ vg/kg AAV8-arRNA | 1 |
| 13-week | 3×10^13^ vg/kg AAV8-arRNA | 1 |

-	Is it possible that the reason for a lack of dose-dependent effect is due to saturation of endogenous ADAR? The text states that there is a dose-dependent increase in AAV transduction rate however there are no statistics on figure 2C and 2D - was this not significant between doses?

*Response: Thanks for your suggestion. Since Fig. 2B was the result of a liver biopsy, we speculated that the lack of dose dependence was due to uneven sampling. We have added statistical analysis in Fig. 1C and Fig. 1D. The statistical results showed that the VCN of AAV and the RNAscope showed significant differences according to the dose of AAV.*

-	With the RNA analysis shown in Figure 3 the authors should check not just for off-target effects but also for decreases in editing of endogenous sites between the control and AAV-treated animals.

1. With the RNA analysis shown in Figure 3 the authors should check not just for off-target effects but also for decreases in editing of endogenous sites between the control and AAV-treated animals.

*Response: Thank you for your suggestion. To address this question, we examined the RNA editing patterns in the livers of four NHPs, including two untreated control groups and two treated groups (receiving 3×10^13^ vg/kg of AAV8-arRNA at 4 weeks and 13 weeks). We retrieved the editing data for non-repetitive editing sites from all 4 animals by combining the data from three technical replicates for each sample. As shown below, there is some degree of variability in the editing of endogenous sites among individuals. In the two control groups, the edited patterns differed between individuals. For instance, some sites showed 100% editing efficiency in Control 1, while Control 2 showed 0% editing efficiency at the same sites. This aligns with the current understanding that RNA editing patterns can vary among individuals (Reviewer Figure 2). Therefore, in evaluating potential off-target effects, we focused on newly emerging editing sites in the treated group. To delve deeper, we calculated correlation coefficients among the four groups for all RNA editing sites. The results indicated that the four groups had similar correlation coefficients (Reviewer Table 2).*

*Reviewer Figure 2.* *Global RNA editing patterns across four groups of NHPs. A total of 2301 sites were analyzed (after filtering out repetitive regions) that had a sequencing depth of at least 50 in all four samples and at least one sample with 3 or more editing reads, encompassing both mock and AAV-injected treatment groups.*

Reviewer Table 2. Correlation coefficient among different groups.

| Comparison | Correlation coefficient (R) |
| --- | --- |
| Control1 vs Control2 | 0.828 |
| Control1 vs 4-week | 0.799 |
| Control1 vs 13-week | 0.844 |
| 4-week vs 13-week | 0.838 |

-	The text in lines 101-104 could be more specific - as only one NHP was kept long term it does not make sense to say "up to 3 months" the authors should be specific about the number of days. Likewise the following sentence is not very clear on why they point out no decrease for 2 biopsies at weeks 2 and 4 but not 13-weeks?

*Response: Thank you for your suggestion. We have unified all descriptions.*

-	For figure 3D, it would be more standard to represent this data as a Volcano plot to see how many statistically significant gene changes there are between control and treated animals. The correlation plot does not allow assessment of how significant the effects on gene expression globally are. It would also be interesting to see whether interferon-stimulated genes are activated due to reprogramming of ADAR and loss of editing at endogenous immunogenic sites (or whether ADAR expression is changed).

*Response: Thank you for your question. To address this concern, we evaluated the liver gene expression variations in four NHPs by RNA-seq. The analysis included two control groups and two treatment groups that received 3×10^13^ vg/kg of AAV8-arRNA for 4 weeks and 13 weeks. By comparing the gene expression differences between the various groups, we observed that more than 1000 genes exhibited significant changes between the control group (Control 1 vs Control 2). Similarly, comparable levels of gene expression differences were also present between the control group and the experimental group (Reviewer Figure 3). This variability could be attributed to individual differences among the NHP. Further investigation revealed that the expression levels of ADAR genes did not exhibit significant differences across the groups.*

*Reviewer Figure 3.* *Genes exhibiting substantial expression differences with Padj<0.01, |log2FC|>2, FPKM>2.*

*Analysis of 218 ISGs reveals distinct patterns among different individuals. We speculate that inter-individual variability might be the cause for the differences observed in gene expression (Reviewer Figure 4). Expanding the sample size for further analysis could potentially lead to more conclusive findings.*

 *Reviewer Figure 4. ISG were retrieved from the GO terms of humans and rhesus monkeys using the AmiGO2 server. Only genes with an FPKM greater than 0.5 in all replicates from 4 animals are shown.*

-	For the optimization of the 5' and 3' lengths in the circ-arRNA, could the authors comment on why these lengths were chosen and why the 5' length needs to be longer than the 3' length.

*Response: Thank you for your suggestion. We chose these lengths based on findings from previous research (PMID: 31308540, PMID: 35145313). In our earlier studies, we found that the length of the 5' end is more important for arRNA editing, and 30 nt to 110 nt represents the range of arRNA lengths that can be effectively edited.*

-	Since the Humanized Hurler syndrome mouse is not used in Figure 4 it would be clearer to move the illustration in part A to Figure 5.

*Response: Thank you for your suggestion. We have made the change as suggested.*

-	The editing percentage in the GM06214 cells is very low compared to other experiments. Could the authors comment on this? Is this due to the cell type or the AAV vector used? For the IDUA enzymatic activity shown it would be helpful to have a WT control to be able to compare how significant this improvement in enzyme function is.

*Response: Thank you for your question. The GM06214 cells are fibroblast, which are not the optimal target cells for AAV-PHP.eB. Therefore, the infection efficiency in these cells is low, and the expression levels of arRNA within the cells are also quite low. This contributes to the low editing efficiency observed. Additionally, we do not have suitable controls available at the moment, and as such, we are unable to assess the extent of recovery in the current setting.*

-	Statistical analysis on figures 4 and 5 is completely absent which drastically limits the interpretation of the findings and how meaningful the observed changes are particularly in the Hurler-syndrome mouse model. This should be added.

*Response: Thank you for your suggestion. We have added the statistical analysis for Fig. 4 and Fig. 5.*

-	No WT (or heterozygous) control is included in the analysis of Humanized Hurler syndrome mouse model which would be very helpful in the interpretation of how significant the improvement in IDUA gene function is relative to WT. While the lack of this does not negate the findings it does limit interpretation. The authors could compare to other data using these assays with WT mice and comment on how substantial the changes are. Furthermore, there is no description of the phenotype of this mutation in mouse - are they sick, is survival affected? While enzymatic activity is improved, does this improve the overall health of the animals? How does this model compare to phenotypes in humans with this mutation?

*Response: Thank you for your suggestion. Since we constructed this mouse for the first time, we placed the CDS region of the human IDUA^W402X^ gene under the control of a strong promoter (CAG promoter) and were successful in integrating this cassette into a mouse with a defective idua^W392X^ gene (see Fig. S5). We observed that this disease model mouse lacked IDUA enzyme activity (as shown in Fig. S5), which is the critical characteristic of Hurler syndrome, leading us to believe that this mouse model is representative of humanized Hurler syndrome. However, we did not employ the same method to construct a mouse model with the WT hIDUA gene as a control, so we did not include a control in Fig. 5 due to the lack of an appropriate control group.*

*To address* *this concern, we used WT C57BL/6 as a control. We compared the GAG content in treated humanized IDUA^W402X^ mice with that in WT C57BL/6 mice. We observed that the GAG content in the treated mice was similar to the levels found in WT C57BL/6 mice, indicating the effectiveness of the treatment (see Reviewer Figure 5).*

*Reviewer Figure 5. Tissue GAG content in the liver after six weeks of infection with AAV-PHP.eB. n ≥ 3.*

*Indeed, this mouse model only contains the humanized IDUA^W402X^ gene and transcript, while the ADAR protein involved in the editing process is endogenous to the mouse and not the human ADAR protein. Therefore, it cannot fully represent a patient with the same mutation, as the expression levels of the target transcript, arRNA, and ADAR protein might differ between the model and a human patient. However, our results indicate that both human-derived HEK293T cells and the mouse model exhibit a similar editing pattern. This leads us to* *hypothesize that the arRNA optimized in vitro can achieve similar effects in animals. This constitutes one of the main conclusions that we have drawn from this animal model.*

-	Is the 2x Circ-arRNA used in Figure 5 using the 2x U6 promoters shown in Fig S7? This should be explicitly stated. There is no interpretation of the comparison of the different circ-arRNAs used in this figure in the text. This should be added to the discussion of this figure.

*Response: Thank you for your question. We used 2×U6 promoters in Figure 5. This detail has been incorporated and discussed within the figure.*

Minor comments:

1. On the first use of an acronym they should be defined - including arRNA, LEAPER and NHP

*Response: Thank you for your suggestion. We have made changes accordingly.*

2. The introduction would benefit from a brief introduction to the LEAPER method.

*Response: Thank you for your suggestion. We have made changes accordingly.*

3. The manuscript should be checked for grammar. Some specific minor corrections noted here:

a. line 54 - remove "a" before the word robust

b. line 71 - change delivered to delivery

c. line 107 - change "to LEAPER" to "of LEAPER"

d. line 172 - "test" to "testing"

e. line 178 - typing error in parallel

f. line 187 - I believe the authors mean systemic not systematic?

g. Line 806 - breaded to bred.

*Response: Thank you for your suggestion. We have made changes accordingly.*

4. What does CRO stand for in the Figure 2 figure legend?

*Response: Thanks for your suggestion. We have changed the description. “Yellow areas are a historical range of health non-human primates.”*

Reviewer #3: RNA editing holds promise as a therapeutic strategy that enables temporary and non-heritable edits. The utilization of systems that deliver a single gRNA to recruit endogenous ADARs has great therapeutical potential. In this study, the authors demonstrate the effectiveness of AAV-delivered circular arRNAs for achieving RNA editing in non-human primates at therapeutic doses. Furthermore, AAV-delivered circular arRNAs in a humanized Hurler syndrome mouse model rescued IDUA enzymatic activity in multiple organs. While this work provides preliminary evidence supporting the feasibility of AAV-based gRNA delivery in therapy and translational research, a number of concerns need to be addressed to substantiate their claims.

*Response: Thank you for your encouraging comments.*

1. The NHP experiments have a key flaw as the authors only tested a single site with a TAG motif, which is known to be the easiest to edit, to showcase the potential of cir-arRNA for RNA editing in the NHP liver. To establish the efficacy of AAV-mediated LEAPER 2.0 for editing in NHP liver, it is crucial for the authors to test additional sites, especially those with non-TAN motifs, to demonstrate that efficient editing can be achieved beyond the TAG motif.

*Response: Thank you for raising this question. As pointed out by Review #3 in the following question, we have observed significant off-target editing at positions 13, 39, 44, and 47. The corresponding motifs at these positions are CAC, TAT, TAG, and AAT. When we consider the data from positions 0 (TAG), 13 (CAC), and 47 (AAT), it becomes evident that efficient editing can occur with motifs that have not only TAN but also CAN and AAN. However, in the case of GAN motifs, LEAPER 2.0 has been reported to struggle in achieving high editing efficiency without the introduction of additional mismatches. It should be noted that the arRNA used in this experiment was designed to target the 0 (TAG) site and was not optimized for positions 13, 39, 44, and 47. This implies that the arRNA has an A/U match at these positions (see Reviewer Figure 6). As it is well established that an A/C mismatch can increase editing efficiency by approximately 2-3 folds than an A/U match, it is conceivable that optimization of arRNA could lead to higher editing efficiency at these positions. This underscores the versatility of LEAPER 2.0, which can be effective for not just TAN motifs but also CAN and AAN motifs.*

*For GAN motif, a previous study by the Thorsten Stafforst group demonstrated that using GCN instead of CCN as the targeting triplet in arRNA could substantially boost the editing efficiency for GAN targets in cells with overexpressed ADAR (PMID: 24744243). Our own* *unpublished data corroborates this finding, even with endogenous ADAR. This suggests a viable approach to target GAN motifs using the LEAPER technology.*

*Reviewer Figure 6. Different motif editing rate of arRNA in NHPs (3×10^13^ vg/kg AAV8-arRNA). Each point indicated a result from a single lobe of the liver; mean ± SD.*

2. From Fig. 1F, it appears that the arRNA shows considerable off-target editing at positions 13, 39, 44, and 47. It would be important to investigate the editing statuses of these sites in the NHP liver. If these sites are also edited in NHP liver, the authors should use a deletion strategy to remove these unwanted edits in vivo. Since deletions generally decrease the dsRNA structure between arRNA and its target, it is crucial to demonstrate that this approach maintains on-target editing while removing off-target edits in this case.

*Response: Thank you for your question. This question posed by Review #3 can be divided into 2 parts.*

*Firstly, it's important to address the off-targets shown in Fig. 1F, especially at positions 13, 39, 44, and 47, which are derived from AAV-infected NHP hepatocytes in an in vitro system. The crucial query is whether this off-target pattern remains consistent between in vitro and in vivo environments. As LEAPER employs endogenous ADAR for RNA editing, the editing pattern is dependent on the expression of ADAR isoforms and the RNA sequences. Comparing in vitro and in vivo counterparts like hepatocytes and liver, their transcriptome profiles are similar. Consequently, the endogenous ADAR isoforms that are expressed are expected to be similar, leading to the premise that the editing pattern between on-target and off-target should be predominantly governed by RNA sequences. This aligns with the observations made in this study. As shown in the figure, (which contains the same data as Fig. 1B/C/F), the off-target pattern is conserved between the in vitro and in vivo settings.*

*Reviewer Figure 7. The same circ-arRNA has the same editing pattern in vitro and in vivo.*

*On another note, there is a concern regarding the introduction of deletions to eliminate off-target editing and its potential impact on the dsRNA structure. The question is whether this approach can effectively eliminate off-target editing while preserving the efficiency of on-target editing. As shown in Fig. 1B, when we first introduced an arRNA that is fully complementary to PPIA mRNA with the exception of a single A/C mismatch at the target site, we observed varying levels of editing at several adenosine sites in the vicinity of the target site. We chose the top 3 sites (5, -23, -28) and deleted them either individually or altogether. The results indicate that the on-target editing efficiency remains unchanged, whereas the off-target editing efficiency at positions 5, -23, and -28 was almost completely eradicated (Fig. 1C). As shown above, the editing at positions 5, -23, and -28 appeared to be nearly eliminated in the NHP liver (Reviewer Figure 7). These results indicate that our U deletion strategy also work in NHP liver, and is capable of removing off-target editing while maintaining on-target efficiency.*

*However, it is crucial to note that the NHP PPIA is a housekeeping gene that does not harbor any disease-causing mutation. In previous studies, we demonstrated that human PPIA can be optimized to remove all off-target editing while maintaining on-target efficiency (PMID: 35145313). Therefore, instead of further optimizing NHP PPIA, we chose to optimize an arRNA targeting human disease-causing G to A mutation, specifically IDUA W402X. When applying LEAPER to IDUA W402X as shown in Fig. 4I, we observed off-targets that are of less concern. Like synonymous mutation in DNA, editing a specific adenine does not change the encoded amino acid regardless of whether editing takes place. Consequently, we did not aim to eliminate all off-targets and left the +7 position unoptimized. Thus, for IDUA^W402X^, arRNA can be optimized to remove all off-target except synonymous positions while retaining on-target efficiency.*

3. Figure 3 requires a more detailed analysis of global off-targets.

a) Editing sites with >5% or 10% editing levels may have phenotypic effects. Thus, the authors need to accurately describe the landscape of off-targets using lower editing level cutoffs.

*Response: The selection of the >5% and <10% cutoffs are aimed at identifying differentially edited sites, as described in the methods section. The 5% cutoff is employed to select authentic editing events where the sequencing depth is relatively low, at a minimum of 50 reads across three replicates. Specifically, 5% is proposed as the lowest detection limit, corresponding to an average of at least 1 edited read for each replicate, totaling at least 3 edited reads in 50 reads across the three replicates (~6%).*

*On the other hand, the 10% cutoff for identifying differential editing is decided by the sequencing depth. For a minimum sequencing depth of 50× and a desired P-value of less than 0.05 (using Fisher’s exact test), and an adjusted P-value (Padj) of less than 0.01 to minimize false positives, the minimum change in editing efficiency needs to be 12% (for Padj < 0.01 is 18%). For a 100x minimum sequencing depth, these values are 6% and 8% respectively. The majority of editing sites typically have lower sequencing depth, so this cutoff, in conjunction with the Padj cutoff, is useful in mitigating sampling error. For the minority of sites with ultra-high sequencing depth, this cutoff is still effective in selecting significantly differentially edited sites, accounting for any amplification bias or imbalance in sampling among replicates.*

*Furthermore, it's important to note that using a 10% difference cutoff with a minimum sequencing depth of 50× is a common parameter used in previous RNA editing studies (PMID: 35256816, PMID: 31308540, PMID: 34980913, PMID: 30692694). In our dataset, approximately 95% of the read depths for the detected A>G editing sites are equal to or less than 50× (see Reviewer Figure 8), making the chosen cutoffs appropriate for the analysis.*

*Reviewer Figure 8.* ***(A)*** *Cumulative plot of raw sequencing depth of all A>G sites detected among 4 animals by RNA-Seq;* ***(B)*** *Simulation of P-value and editing efficiency difference cutoffs under 50× and 100× sequencing depth with Fisher’s exact test for selection of significantly differentially editing sites.*

b) The method section for off-target analysis is not clear. The authors need to define how a site is considered an off-target. Additionally, although the method section describes the analysis of differentially edited sites, there are no related results in the results section.

*Response: We have updated the methods section to clarify the procedure used to identify off-target sites. The changes have been highlighted in red.*

*[For each comparison, the RNA editing level of the control group was used as a baseline, and global targets in the treatment group were ascertained by subtracting variants in the control group. This helped in identifying potential off-target sites, taking into account the consistency across all three replicates in both groups. Variants with fewer than 50 reads in total or with an editing level of <5% in all the comparison groups were excluded. The shared global editing sites were assessed using Pearson’s correlation coefficient analysis. For the differential editing of native A-to-I editing sites, global targets with consistency across all three replicates of each group and were covered by more than 50 reads in total were selected. This was done after recognizing the reference strand and combining the data from replicates. The sites were then subjected to pairwise comparison using Fisher’s exact test, with P values adjusted using the Benjamini-Hochberg method. Sites with an adjusted P value of less than 0.01 and an absolute difference in editing level of >10% in either direction were categorized as differentially edited* *sites. The novel sites that were detected only in the treatment group and were consistent across all 3 replicates in both treated animals were considered as potential ‘off-target’ sites. These were compared to those in one untreated animal (Control 1). These potential ‘off-target’ sites were further analyzed through sequence homology analysis by retrieving 100 nt upstream and downstream flanking sequences from the respective overlapping transcript or genomic region. This allowed for an analysis of their similarity and duplex structure in relation to the arRNA sequence.]*

*The findings corresponding to these changes are depicted in Fig. 3E and Fig. S4.*

c) A comparison of editing levels of individual sites between control and edited NHP samples is necessary to estimate the impact of AAV-delivered arRNA on the global editing landscape.

*Response: Thank you for your suggestion. To address this question, we analyzed the RNA editing patterns in the liver of four NHPs, including two untreated and two treated groups (with 3×10^13^ vg/kg AAV8-arRNA, at 4 weeks and 13 weeks). We combined all three technical replicates of each sample to retrieve the editing at non-repetitive common editing sites from all 4 animals. As shown below, the editing at endogenous sites was correlated with individual animals. In the two control groups, there were differences in editing patterns between* *individuals. For instance, at certain positions where the editing efficiency in Control 1 is 100%, the editing efficiency in Control 2 is 0 (see Reviewer Figure 9). These data demonstrate that global RNA editing varies among individuals. To further investigate this question, we calculated correlation coefficients between these four groups for all RNA editing sites. The results showed that the four groups had comparable correlation coefficients (see Reviewer Table 3).*

*Reviewer Figure 9. Global RNA editing patterns among four groups of NHPs. Totally 2301 sites (after filtering repetitive regions) with all four samples depth >= 50 and at least one sample with >=3 editing reads from both mock and AAV-injected treatment groups.*

Reviewer Table 3. Correlation coefficient between different groups.

| Comparison | Global RNA editing correlation coefficient (R) |
| --- | --- |
| Control1 vs Control2 | 0.828 |
| Control1 vs 4-week | 0.799 |
| Control1 vs 13-week | 0.844 |
| 4-week vs 13-week | 0.838 |

d) Further analyses are needed for the off-target sequences to determine possible reasons. For example, investigating the sequence similarity among the off-target regions and examining if short duplex formation occurs between arRNA and off-target sequences.

*Response: A total of 18 non-redundant sites were detected as potential ‘off-target’ sites, from two AAV-treated animals in contrast to the mock animal. These sites were located in the UTR* *or coding regions of 17 genes. We used BEDtools (v2.28.0) to extract sequences flanking 100 nt upstream and downstream from these potential off-target sites, and this was done from the relevant genomic and transcriptomic regions (if the sites were located within a transcript). These sequences were then subjected to a sequence homology analysis in relation to the arRNA sequence by employing the BLAST program (v2.5.0+) using default parameters. Since no substantial hits were found, it appears that these sites seem unlikely to be arRNA-related off-target events. Further analysis using RNAhybrid revealed that these sites could not form effective duplexes with the arRNA, and the calculated MFE (Minimal Free Energy) values were higher than the MFE of the arRNA and target sites. This information is presented in Fig S4.*

4. Fig. 4A: There is no description of the transgenic mice used. The authors should provide information on the promoter used and the expression profile of the hIDUAw402x gene, and whether it is similar to the original gene.

*Response: Thank you for your suggestion. Since we constructed this mouse for the first time, we placed the CDS region of the human IDUA^W402X^ gene under the control of a strong CAG promoter. This construct was successfully integrated into the genome of a mouse harboring a defective idua^W392X^ gene (Fig. S5). We have incorporated this information in Fig. S5 for further clarification.*

5. Fig. 5. The authors should measure the IDUA enzymatic activity and GAG content of wild-type mice as controls. This will provide a baseline for evaluating the extent to which RNA base editing rescues these molecular markers.

*Response: To address this issue, we used WT C57BL/6 as controls and measured the GAG content in both the treated humanized IDUA^W402X^ mice and the WT C57BL/6 mice. We found that the GAG content in the treated mice was comparable to that in the WT C57BL/6 mice, indicating the effectiveness of the treatment, as shown in Reviewer Figure 10.*

*Reviewer Figure 10. Tissue GAG content in the liver after AAV-PHP.eB infection 6 weeks. n ≥ 3.*

6. It is crucial for the authors to show that arRNA-based RNA editing rescues the phenotype of the disease model. Since IDUA mutant mice are known to exhibit bone abnormalities, craniofacial abnormalities, and reduced lifespan, the authors should provide evidence that RNA editing leads to the rescue of these phenotypes. Additionally, histopathological evidence of reduced lysosomal storage in various tissues in the edited samples should be presented.

*Response: We observed that this disease model mouse lacks IDUA enzyme activity (Fig. S5), which is a crucial indicator of Hurler syndrome, leading us to believe that this mouse model effectively represents a humanized Hurler syndrome. It's important to note that monitoring the complications associated with Hurler syndrome requires long-term observation, potentially spanning 1-2 years, which is beyond the scope of this study. Our primary objective was to ascertain whether human transcripts can be effectively edited by LEAPER in animal models. Our findings indicate that LEAPER can efficiently edit human transcripts in animals, and restore protein function, which brings us a step closer to potential clinical application.*

Minor comments

1. Figure 3D. Please label the differentially expressed genes and analyze the functions of the upregulated and downregulated genes, respectively.

*Response: Thank you for your suggestion. To address this question, we analyzed the liver gene different expression profiles of four NHPs by RNA-seq, including two untreated and two treated* *groups (3×10^13^ vg/kg AAV8-arRNA, at 4 weeks and 13 weeks). We compared the differential gene expression between different groups and observed that more than 1,000 genes exhibited significant changes when comparing the control group (Control 1 vs Control 2). Similarly, a comparable level of differential gene expression was also observed between the control group and the experimental group (Reviewer Figure 11). This could be attributed to the intrinsic variations among individual NHPs.*

*Reviewer Figure 11. Significant different expression genes with Padj<0.01, |log2FC|>2, FPKM>2.*

2. RNA-seq data generated in this study should be deposited and released in a publicly accessible repository for transparency and reproducibility.

*Response: Thank you for your suggestion. We have uploaded the data in public database.*

**Second round of review**

**Reviewer 1**

The authors addressed all my comments. The manuscript has significantly improved and I have no further comments.

**Reviewer 2**

The authors have substantially improved the manuscript and I am satisfied with the majority of responses to my concerns. I believe that several of the reviewer figures should be included in the manuscript to assist other readers in interpretation of the results, namely reviewer Figures 2 and 3 which addressed questions from myself and Reviewer 3.

Although the editing analysis shows drastic inter-individual variability, there are some sites that are consistent across all individuals in both treatment and control groups and the lack of change in these in encouraging. Without more samples it is difficult to interpret beyond this, although the difference between individuals at sites with relatively high depth is interesting.

The additional statistics added to the figures is still limited in how they can be interpreted because only comparison to control groups is shown. Pairwise comparisons between treatment groups would allow assessment of which guide designs/conditions are better. This is particularly important for Figure 4C-D.

**Reviewer 3**

The authors have addressed most of my concerns, and I only have two remaining comments.
Regarding the quest to demonstrate that arRNA-based RNA editing rescues the phenotype of the disease model. >> I understand that monitoring the complications associated with Hurler syndrome requires long-term observation, potentially spanning 1-2 years, which may not be easy. However, this study should at least include some simple experiments, such as histopathological evidence of reduced lysosomal storage in various tissues. In the authors' 2022 Nature Biotech paper, the editing efficiency in a mouse Hurler syndrome model has already been demonstrated. Therefore, the objective here is not only to show that it can edit another mouse model with transgenic human transcript, but more importantly, to provide evidence that it can rescue the phenotypes.
Regarding the previous question "A comparison of editing levels of individual sites between control and edited NHP samples is necessary to estimate the impact of AAV-delivered arRNA on the global editing landscape." >> Instead of using non-repetitive common editing sites for analysis, I suggest that the authors use all editing sites identified in any of the animals for analysis. This approach could provide an unbiased assessment of the impact of AAV-delivered arRNA on the global editing landscape.

**Authors Response**

**Point-by-point responses to the reviewers’ comments:**

Reviewer #1:

The authors addressed all my comments. The manuscript has significantly improved and I have no further comments.
*Response: We appreciate your supportive remarks.*

Reviewer #2:

The authors have substantially improved the manuscript and I am satisfied with the majority of responses to my concerns. I believe that several of the reviewer figures should be included in the manuscript to assist other readers in interpretation of the results, namely reviewer Figures 2 and 3 which addressed questions from myself and Reviewer 3.
*Response: We appreciate your supportive remarks.*

Although the editing analysis shows drastic inter-individual variability, there are some sites that are consistent across all individuals in both treatment and control groups and the lack of change in these in encouraging. Without more samples it is difficult to interpret beyond this, although the difference between individuals at sites with relatively high depth is interesting.
*Response: Thank you for your understanding. Due to ethical considerations, our present research is constrained to a limited number of monkeys. If we aim to undertake a more extensive investigation of this issue, we will need a larger quantity of experimental animals.
The additional statistics added to the figures is still limited in how they can be interpreted because only comparison to control groups is shown. Pairwise comparisons between treatment groups would allow assessment of which guide designs/conditions are better. This is particularly important for Figure 4C-D.*
*Response: We appreciate your suggestion. After reviewing the data, we have rephrased it and determined that the conclusion remains unchanged. Additionally, we have made modifications to the relevant figure.*

Reviewer #3:

The authors have addressed most of my concerns, and I only have two remaining comments. Regarding the quest to demonstrate that arRNA-based RNA editing rescues the phenotype of the disease model. >> I understand that monitoring the
complications associated with Hurler syndrome requires long-term observation, potentially spanning 1-2 years, which may not be easy. However, this study should at least include some simple experiments, such as histopathological evidence of reduced lysosomal storage in various tissues. In the authors' 2022 Nature Biotech paper, the editing efficiency in a mouse Hurler syndrome model has already been demonstrated. Therefore, the objective here is not only to show that it can edit another mouse model with transgenic human transcript, but more importantly, to provide evidence that it can rescue the phenotypes.
*Response: We’re grateful for your suggestion. Given that Hurler's syndrome primarily manifests as metabolic abnormalities in the liver, we conducted a histopathological examination of the liver. The findings revealed that mice treated with arRNA displayed improvement, exhibiting characteristics similar to those of wild-type mice.*

*Response Figure 1. Hematoxylin-Eosin staining of mouse livers in different groups. Red arrows indicate foamy macrophages in the tissue due to GAGs accumulation. Scale bar = 50 μm.
Regarding the previous question "A comparison of editing levels of individual sites between control and edited NHP samples is necessary to estimate the impact of AAV- delivered arRNA on the global editing landscape." >> Instead of using non-repetitive common editing sites for analysis, I suggest that the authors use all editing sites identified in any of the animals for analysis. This approach could provide an unbiased assessment of the impact of AAV-delivered arRNA on the global editing landscape. Response: We appreciate your suggestion. To address this query, we conducted an analysis of liver RNA editing patterns in four NHPs, comprising two untreated and two treated groups (at dosages of 3×1013 vg/kg AAV8-arRNA for 4 weeks and 13 weeks). The editing sites from all 4 animals was retrieved by combining all three technical replicates of each sample’s RNA-Seq data. We considered variants that were covered by no fewer than 50 reads in total, with at least 3 supporting reads showing editing in at least one group. The shared global editing sites were evaluated using Pearson's correlation coefficient analysis in pairwise. As shown below, the editing of endogenous sites exhibited a* *correlation with the specific animal. Within the two control groups, the edited pattern displayed variations between individual subjects. We have included these data in the supplementary section.*

*Response Figure 2. Comparison of global editing landscape in different groups.*

**Third round of review**

**Reviewer 2**

The authors have satisfactorily addressed my comments.

**Reviewer 3**

The authors addressed all my comments.
